# IL-17 Aggravates *Pseudomonas aeruginosa* Airway Infection in Acute Exacerbations of Chronic Obstructive Pulmonary Disease

**DOI:** 10.3389/fimmu.2021.811803

**Published:** 2022-01-13

**Authors:** Fengming Ding, Lei Han, Qiang Fu, Xinxin Fan, Rong Tang, Chengjian Lv, Yishu Xue, Xue Tian, Min Zhang

**Affiliations:** ^1^ Department of Respiratory and Critical Care Medicine, Shanghai General Hospital, Shanghai Jiao Tong University School of Medicine, Shanghai, China; ^2^ Department of Tuberculosis, Fuzhou Pulmonary Hospital of Fujian Province, Fuzhou, China; ^3^ Department of Clinical Laboratory, Shanghai General Hospital, Shanghai Jiao Tong University School of Medicine, Shanghai, China

**Keywords:** COPD, IL-17, *Pseudomonas aeruginosa*, cytokine profile, lung function

## Abstract

*Pseudomonas aeruginosa* airway infection increases risks of exacerbations and mortality in chronic obstructive pulmonary disease (COPD). We aimed to elucidate the role of IL-17 in the pathogenesis. We examined the expression and influences of IL-23/IL-17A in patients with stable COPD (*n* = 33) or acute COPD exacerbations with *P. aeruginosa* infection (*n* = 34). A mouse model of COPD (C57BL/6) was used to investigate the role of IL-17A in host inflammatory responses against *P. aeruginosa* infection through the application of IL-17A–neutralizing antibody or recombinant IL-17A. We found that *P. aeruginosa* infection increased IL-23/17A signaling in lungs of both COPD patients and COPD mouse models. When COPD mouse models were treated with neutralizing antibody targeting IL-17A, *P. aeruginosa* induced a significantly less polymorphonuclear leukocyte infiltration and less bacterial burden in their lungs compared to those of untreated counterparts. The lung function was also improved by neutralizing antibody. Furthermore, IL-17A-signaling blockade significantly reduced the expression of pro-inflammatory cytokine IL-1β, IL-18, TNF-α, CXCL1, CXCL15 and MMP-9, and increased the expression of anti-inflammatory cytokine IL-10 and IL-1Ra. The application of mouse recombinant IL-17A exacerbated *P. aeruginosa*-mediated inflammatory responses and pulmonary dysfunction in COPD mouse models. A cytokine protein array revealed that the expression of retinol binding protein 4 (RBP4) was down-regulated by IL-17A, and exogenous RBP4-recombinant protein resulted in a decrease in the severity of *P. aeruginosa-*induced airway dysfunction. Concurrent application of IL-17A-neutralizing antibody and ciprofloxacin attenuated airway inflammation and ventilation after inoculation of *P. aeruginosa* in COPD mouse models. Our results revealed that IL-17 plays a detrimental role in the pathogenesis of *P. aeruginosa* airway infection during acute exacerbations of COPD. Targeting IL-17A is a potential therapeutic strategy in controlling the outcomes of *P. aeruginosa* infection in COPD patients.

## Introduction

*Pseudomonas aeruginosa* is a common cause of bacterial infection in Chronic Obstructive Lung Disease (COPD), which can be isolated from sputum samples of 4%-15% of adult COPD patients (1, 2). COPD patients in whom *P. aeruginosa* can be cultured from the airways had a markedly increased risk of exacerbations and mortality (3), which are more likely to be seen in patients who have received recent antibiotic therapy, and those who require mechanical ventilation (4). In most cases, the carriage of a strain lasts for a short time, however a subset of patients can be persistently colonized with *P. aeruginosa* for several years (5). The main factors contributing to the pathogenicity of *P. aeruginosa* are the type III secretion-linked cytotoxicity and production of quorum-sensing regulated virulence factors, such as pyocyanin, alkaline proteases, and elastase (6). Several conserved microbial structures have been implicated in activating the host immune responses through the cell surface and endosomal Toll-like receptors (TLRs), such as TLR4 and TLR5 (7, 8).

However, the immune responses to *P. aeruginosa* infection are not always protective, and an excessive host inflammatory response can cause lung injury (9). For example, neutrophils are crucial for the clearance of bacterial pathogens, but persistent neutrophil recruitment and excessive release of proteases from neutrophils, such as neutrophil elastase (NE) and matrix metalloproteinase-9 (MMP-9), leads to excessive extracellular matrix (ECM) degradation and tissue damage (10). Neutrophils degranulation also releases excessive oxidants, including hydrogen peroxide and hydroxyl radicals that attack host tissues (11). The ability of *P. aeruginosa* to activate the NLRC4 inflammasome-mediated production of IL-1β and IL-18 is responsible for a substantial amount of the pathology associated with acute pneumonia, and inhibition of IL-1β, caspase-1, IL-1R, and IL-18R limits pathological consequences of infection and improves bacterial clearance (12, 13). Therefore, rapid resolution of *P. aeruginosa* infection-induced inflammation is important for reducing the lung injuries, which involves the balance of pro- and anti-inflammatory immune responses including the expression of cytokines and chemokines. The IL-17 family of cytokines is one of such group that can influence the balance of immune responses.

The IL-17 cytokine family consists of six related proteins, IL-17A-F (14). IL-17A is the prototypical member of this family, and signals through a multimeric receptor consisting of IL-17RA and IL-17RC, along with IL-17F that sharing 50% homology to IL-17A. Other family members signal through receptor complex, sharing the common chain with IL-17RA (15). Although Th17 cells are a major source of IL-17 cytokines which are induced by IL-23, they can also be produced by innate immune cells, including dendritic cells (DCs), macrophages, γδT cells, and type 3 innate lymphoid cells (16). The role of IL-17 family cytokines in COPD has emerged rapidly in the last decade. Both stable COPD and acute exacerbations of COPD (AECOPD) have been associated with elevated levels of IL-17A or IL-17A-producing cells in clinical studies (17). IL-17 receptor signaling was shown to be critical for the development of lung inflammation and emphysema in a study using murine models of cigarette smoke-induced COPD, suggesting a critical role for IL-17 in chronic lung injury associated with chronic airway inflammation (18).

In infectious diseases, IL-17 has been shown to play both protective and pathogenic roles. Proper IL-17 signaling enhances the immunity that protects the host from bacterial, fungal and viral invasion (19–21). It is reported that IL-17 rather than antibody is a key element in host defense against chronic pulmonary infection with *P. aeruginosa* (22). In contrast, aberrant IL-17 signaling can lead to excess pulmonary inflammation which can lead to immunopathology and inflammation-induced tissue destruction, such as acute respiratory distress syndrome (ARDS) (23). IL-17A also plays a detrimental role in the pathogenesis of other bacterial mucosal disorders such as *P. aeruginosa* keratitis (24). Therefore, IL-17 are not only involved in the host defense against infection but also in the tissue damage caused by inflammation.

Given the two sides of IL-17 in mucosal immunity, we are interested in understanding the expression of IL-17 signaling and its role in AECOPD with *P. aeruginosa* infection. In this study, we found blockade of IL-17 signaling significantly attenuated the severity of *P. aeruginosa* airway infection by suppressing infection-induced cytokines, suggesting IL-17A may be used as a target of adjunctive therapy, in combination with antibiotics, to treat AECOPD patients with *P. aeruginosa* infection.

## Materials and Methods

### Paticipants and Clinical Variables

Patients with AECOPD with *P. aerugionosa* infection were recruited between August 2020 to July 2021 from two centers of Shanghai General Hospital (Shanghai, China). One center was at north Hongkou Campus in the urban area, and the other center was at south Songjiang Campus in the suburban area. Chest HRCT scans and spirometries were performed in suspected patients with chronic coughing, expectoration, and trachypnea, and diagnosis of COPD was confirmed according to the Global Initiative for Chronic Obstructive Lung Disease (GOLD) guidelines (25). AECOPD is defined as an acute, sustained (> 48 hours) worsening of respiratory symptoms, such as cough, sputum production, and/or dyspnea, which is beyond normal day-to-day variations, and leads to a change in medication. All these enrolled AECOPD patients presented with purulent sputum at the time of admission into hospital. Culture and subsequent detection of microbial isolates were performed in sputum samples or bronchoalveolar lavage fluid (BALF), and *P. aeruginosa* was positive in the samples of all recruited patients. The density of *P. aeruginosa* in sputum sample was more than 10^7^ CFU/mL, and in BALF sample was more than 10^4^ CFU/mL. These patients didn’t have history of *P. aeruginosa* isolation in previous cultures of lower respiratory tract samples. Patients with malignancy, bronchiectasis, significant immuno-deficiencies, or co-colonized with other detected pathogens (eg. *Staphylococcus aureus*, *Haemophilus influenza* and *Tuberculous mycobacteria*) were excluded. Controls were from a collection of stable COPD patients who had no pathogen found in either sputum or BALF samples. Written informed consent was obtained from all subjects, and this study was proved by the Ethics Committee of Shanghai General Hospital, and performed in accordance with relevant guidelines and regulations.

Characteristics of participants were collected as follows: anthropometric data (age, gender, body mass index and smoking history); spirometry results [percentages of predicted values of forced expiratory volume in one second (FEV_1.0_), forced vital capacity (FVC), the ratio of FEV_1.0_/FVC, peak exiratory flow (PEF), maximal mid-expiratory flow (MMEF), and forced oscillation test (FOT)]; blood indicators (neutrophil absolute counts, neutrophils%); neutrophil counts and cytokines concentrations (IL-17A, IL-23) in BALF.

### Animals and Establishment of COPD Model

Specific pathogen-free male C57BL/6 mice (8-12 weeks of age, 18-22 g) were obtained from Shanghai SLAC Laboratory Animal Co. Ltd (Shanghai, China), and were housed under specific pathogen-free conditions with standard mouse chow (#7001, Harlan Teklad). Animals were exposed to ozone produced from an ozoniser (Model 500 Sander Ozoniser, Germany), mixed with air, for 3 h at a concentration 2.5 parts per million (ppm) in a sealed perspex container, twice a week for 6 weeks. Ozone concentration was continuously monitored with an ozone probe (ATi Technologies, Ashton-U-Lyne, UK). Lung function were measured 24 hours after the last exposure to confirm the COPD model (26, 27). Mice exposed to air were used as controls. All animal procedures were performed in compliance with guide for the care and use of laboratory animals (28) and were approved by the institutional animal care and use committee of Shanghai Jiao Tong University.

### Intratracheal Inoculation With Agar-Entrapped *P. aeruginosa*

After **exposure to ozone or air**, mice were inoculated with agar-entrapped *P. aeruginosa* as previously described (29). Briefly, a virulent laboratory strain PAO1 was embedded in the agarose beads at a final concentration of 2.0 × 10^6^ CFU/mL phosphate buffer solution (PBS). Mice were anesthetized with an i.p. injection of pentobarbital sodium (50 mg/kg) before surgical procedures. The trachea was cannulated, and 50 μL of PAO1-laden agarose beads solution was introduced into the lung. Mice were monitored, and allowed to recover until euthanized by CO_2_ asphyxiation. Mortality rate related to the procedure was 1%.

### Administration of Neutralizing Antibody, Recombinant Proteins and Ciprofloxacin

To apply neutralizing antibody or recombinant proteins, mice were intraperitoneally injected with IL-17A–neutralizing antibody (2 mg/kg, 0.1 ml; R&D Systems, Minneapolis, MN, USA), recombinant mouse (rm)-IL-17A (1.6 mg/kg, 0.1 ml; R&D Systems, Minneapolis, MN, USA) or recombinant mouse retinol binding protein 4 (RBP4, 5 μg/kg, 0.1 ml; Abcam, Cambridge, MA, USA) 4 h before the inoculation with *P. aeruginosa* intrabronchially. To explore the clinical use of anti–IL-17 treatment, anti–IL-17 antibody (2 mg/kg, 0.1 ml) was administered intraperitoneally starting 16 h after *P. aeruginosa* inoculation and continuing every 4 h after initial treatment. Meanwhile, ciprofloxacin (5 mg/kg, Sangon Biotech, Shanghai, China) was administered orally at the same starting point with anti–IL-17 antibody, and continuing every 12 hours after the initial dose.

### Spirometry for COPD Mouse Models Using the Forced Manoeuvres System

Twenty-four hours after the last challenge, mice were anesthetized with an anesthetic solution containing tiletamine hydrochloride and zolazepam hydrochloride (25 mg/kg, Virbac S. A., France) and xylazine hydrochloride (10 mg/kg, Chang Sha Best Biological Technology Institute Co., Ltd, Hunan, China) *via* intraperitoneal injection. Mice were tracheostomized, cannulated endotracheally, and ventilated with a pneumotachograph connected to a transducer (EMMS, Hants, UK) at 250 breaths/min and tidal volume of 250 ml. To mimic clinical spirometry, the lungs of mice were inflated to a set tracheal pressure, and then exposed to a large negative pressure reservoir, forcing the mouse to exhale as quickly as possible. Variables, including the FVC, FEV_50_, MMEF, FEF_50_ and FEF_75_, were calculated.

### Lung Histology and Immunofluorescence

The right low lobes were embedded in paraffin, cut into slices (5 μm), and stained with hematoxylin and eosin. Infiltrate scores were assigned to the infected tissues for the assessment of peribronchial infiltrate severity in a blinded fashion (30). For immunohistochemistry, 6 μm thick sections were cut, mounted to poly-L-lysine–coated glass slides, and blocked with PBS containing 2% BSA for 1 h at room temperature. Sections were then incubated with primary anti-mouse IL-17RA antibody (1:50; Santa Cruz Biotechnology, Dallas, TX, USA) and anti-mouse Ly-6G antibody (1: 100; Cell Signaling Technology, Beverly, MA, USA), followed by the secondary antibodies, including FITC-conjugated anti-rabbit IgG and Cy3-conjugated anti-mouse IgG (1:500; Jackson ImmunoResearch Laboratories, West Grove, PA, USA). The sections were finally counterstained with 4′,6-diamidino-2-phenylindole to visualize the nuclei. Controls were similarly treated with corresponding IgG from the same animal as the primary antibody.

### Quantification of *P. aeruginosa* Burden, and Myeloperoxidase Concentration

The remaining right lobes were excised aseptically and homogenized in 0.5 mL of normal saline. Minced lung tissues were quantitatively cultured by serial dilution on LB agar plates overnight at 37°C. The homogenates were further lysed for myeloperoxidase (MPO) measurement. MPO activities were determined using a MPO Activity Assay kit (Nanjing Jiancheng Bioengineering Institute, Nanjing, China).

### Semiquantitative and Quantitative PCR

Lungs were lysed in TRIzol and total RNA was extracted. For semiquantitative PCR, cDNA was amplified with DreamTaq Green DNA polymerase (Takara Bio., Dalian, Liaoning, China). PCR products were subjected to electrophoresis on 2% agarose gels containing ethidium bromide. For quantitative PCR (q-PCR), cDNA was amplified using a StepOnePlus™ Real-Time PCR Systems with SYBR Green PCR Master Mix (Applied Biosystems, University Park, IL, USA). Data were analyzed by using the 2^-ΔΔCt^ method with β-actin as the internal control. The primers used in this study are listed in **Table S1**.

### Western Blot, ELISA and Cytokine Array

Proteins from lung tissues were extracted using a Nuclear and Cytoplasmic Protein Extraction kit (Beyotime Institute of Biotechnology, Haimen, Jiangsu, China) and protein concentrations were evaluated using a BCA protein assay. For Western blot analysis, A total of 24 μg aliquots of protein samples mixed with a loading buffer were separated by 10% SDS−PAGE and transferred onto polyvinylidene fluoride membranes. The membranes were blocked with 5% milk and subsequently incubated with primary and secondary Abs. The protein bands were illuminated using the enhanced chemiluminescence method (GE Healthcare Life Sciences, Little Chalfont, Buckinghamshire, UK), and quantified by densitometry analyses using ImageJ software version 1.8.0. The primary Abs used included anti-IL-23 (Boster Biological Technology, Wuhan, Hubei, China), anti-IL-23R (Proteintech Group, IL, USA), anti-IL-17A (Proteintech Group, IL, USA), anti-IL-17RA (Servicebio Biological Technology, Wuhan, Hubei, China), anti-RBP4 (R&D Systems, Minneapolis, MN, USA), and anti-β-actin (BioTNT, Shanghai, China). ELISA and Cytokine array (Proteome Profiler Array Mouse XL Cytokine Array Kit; R&D Systems, Minneapolis, MN, USA) were performed following manufacturer’s protocols.

### Statistical Analysis

SAS version 8.1 statistical software (SAS Institute, Inc., Cary, NC, USA) was used for data analysis. Normally distributed quantitative variables were analyzed using *t*-tests, and non-normally distributed variables were analyzed using Mann–Whitney U test. Qualitative variables were analyzed using the chi-squared test. *P* values < 0.05 were considered statistically significant.

## Results

### BALF IL-23/IL-17A Levels Were Elevated in *P. aeruginosa*-Infected COPD Patients

**Figure 1:**
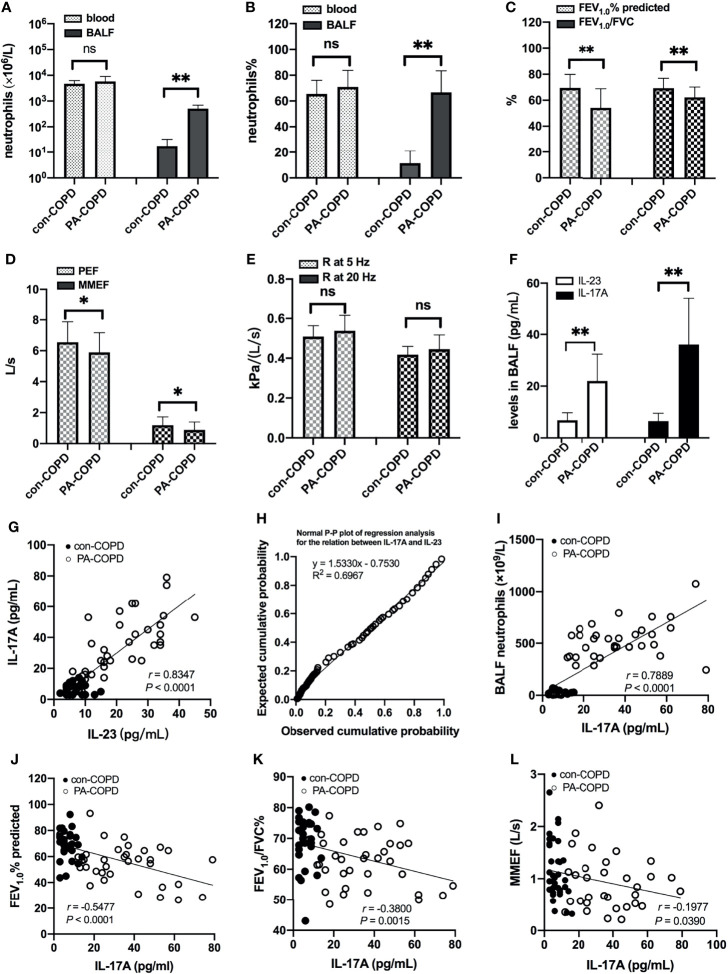
Neutrophils, lung function and BALF IL-23/IL-17A levels between patients with stable COPD (con-COPD, *n* = 33) and patients with *P. aeruginosa*-infected COPD (PA-COPD, *n* = 34). PA-COPD patients had higher absolute and percent numbers of neutrophils in BALF, but not in blood **(A, B)**. Forced expiratory volums **(C)**, expiratory flows **(D)** and forced oscillation tests **(E)** were compared between con-COPD and PA-COPD patients. IL-23 and IL-17A levels were significantly elevated in patients with PA-COPD **(F)**. Data were presented as mean ± SD. *P < 0.05, **P < 0.01, ns, non-significant. Correlation analysis showed that there was a notably positive correlation between the two cytokines **(G)**, and further regression analysis revealed that IL-23 might be an important factor contributing to elevation of IL-17A **(H)**. There was also a significantly positive correlation between IL-17A levels and absolute neutrophil numbers in BALF **(I)**. The spirometry results, including FEV_1.0_% predicted **(J)**, FEV_1.0_/FVC% **(K)** and MMEF **(L)** were negatively correlated with the levels of IL-17A. BALF, bronchoalveolar lavage fluid; COPD, chronic obstructive pulmonary disease; IL, interleukin; FEV_1.0_, forced expiratory volume in one second; FVC, forced vital capacity; MMEF, maximal mid-expiratory flow.

A total of 67 COPD patients were enrolled in the study, including 33 patients with stable COPD (con-COPD group) and 34 patients with *P. aeruginosa*-infected AECOPD (PA-COPD group). The demographic and clinical characteristics of the participants are presented in **Table 1**. There was no significant difference of age, gender, body mass index, pack-years of smoking and baseline lung function between the two groups. Many AECOPD patients received antibiotics before collecting the samples. To avoid the impact of antibiotics on our results, we included some control stable COPD patients who also received antibiotics for infection of other tissues, such as urinary tract and mucous membrane. No significant difference in antibiotic treatment was observed between con-COPD group and PA-COPD group in this study. Compared with con-COPD patients, PA-COPD patients had higher absolute and percent numbers of neutrophils in BALF, but not in blood (**Figures 1A, B**). Spirometry results showed that PA-COPD patients had significantly more severe airflow obstruction and small airway dysfunction during exacerbations than con-COPD patients (**Figures 1C-E**). Significantly higher IL-23 and IL-17A levels were observed in the BALF of PA-COPD patients (**Figure 1F**), and there was a notable positive correlation between levels of the two cytokines (*r* = 0.8347, *P* < 0.0001; **Figure 1G**). Further regression analysis revealed that IL-23 might be an important factor contributing to elevation of IL-17A (R^2^ = 0.6967, regression coefficient =1.5330; **Figure 1H**). IL-17A levels also showed a significantly positive correlation with the levels of absolute neutrophil numbers in BALF (*r* = 0.7889, *P* < 0.0001; **Figure 1I**), and a negative correlation with the spirometry results (FEV_1.0_% predicted, *r* = -0.5477, *P* < 0.0001, **Figure 1J**; FEV_1.0_/FVC%, *r* = -0.3800, *P* = 0.0015, **Figure 1K**; MMEF, *r* = -0.1977, *P* = 0.0390, **Figure 1L**).

**Table 1:** Baseline characteristics of COPD patients with or without *P. aeruginosa* infection.

Characteristic	Con-COPD (n = 33)	PA-COPD (n = 34)	Statistic value^*^	*P*-value
Age, years	70.7 (10.4)	76.3 (16.3)	1.95	0.0548
Male, *n* (%)	29 (87.9)	24 (70.6)	3.03	0.0818
BMI, kg/m^2^	22.3 (3.4)	21.2 (3.4)	1.32	0.1925
Smoking history, pack-years	26.8 (11.8)	32.1 (16.6)	1.51	0.1347
Previous exacerbations, *n* ^**^	2.7 (0.7)	3.0 (0.9)	1.8	0.07
mMRC dyspnea scores, n^***^	2.6 (0.6)	2.8 (0.7)	1.2	0.24
GOLD group	D	D	/	/
spirometric GOLD grade	2	2	/	/
Baseline lung function				
FEV_1.0_% predicted	69.5 (10.5)	65.1 (8.8)	1.85	0.0685
FVC% predicted	81.7 (11.5)	77.2 (7.7)	1.89	0.0636
FEV_1_/FVC (%)	69.2 (7.7)	67.7 (4.3)	0.99	0.3240
PEF, L/s	6.5 (1.3)	5.9 (2.0)	1.61	0.1117
MMEF, L/s	1.19 (0.53)	1.22 (0.70)	0.60	0.5529
R at 5 Hz (R_5_), kPa/(L/s)	0.51 (0.05)	0.49 (0.06)	1.76	0.0825
R at 20 Hz (R_20_), kPa/(L/s)	0.42 (0.04)	0.40 (0.04)	1.74	0.0852
R_5_-R_20_, kPa/(L/s)	0.091 (0.02)	0.086 (0.02)	0.97	0.3368
Antibiotic treatment^****^				
Fluoroquinolone, *n* (%)	7 (21.2)	14 (41.2)	3.10	0.0782
β-lactam, *n* (%)^*****^	4 (12.1)	10 (29.4)	3.03	0.0818
Aminoglycoside, *n* (%)	1 (3.0)	4 (11.8)	1.85	0.1738

Data are presented as median (SD) unless otherwise indicated.

Con-COPD, control COPD group (patients with stable COPD); PA-COPD, P. aeruginosa-infected COPD group (patients with acute exacerbations of COPD with P. aeruginosa infection); BMI, body mass index; FEV1.0, forced expiratory volume in one second; FVC, forced vital capacity; PEF, peak exiratory flow; MMEF, maximal mid-expiratory flow.

*Quantitative variables were analyzed using t-tests, and qualitative variables were analyzed using the chi-squared test.

**COPD exacerbations in the past year.

***mMRC (Modified Medical Research Council) dyspnea scores.

****Antibiotic treatment in 7 days prior the study.

*****β-lactams included β-lactamase inhibitors.

### IL-23/IL-17 Axis Signaling Was Increased by *P. aeruginosa* Infection in COPD Mouse Models

**Figure 2:**
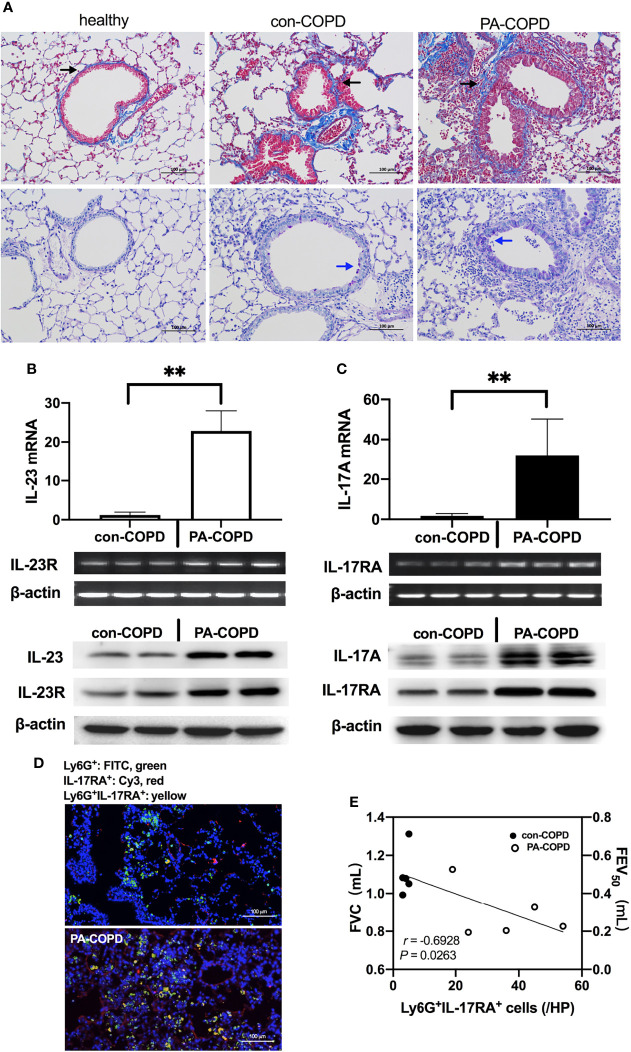
*P. aeruginosa* infection increased IL-23/IL-17 axis signaling in the lungs of COPD mouse models. C57BL/6 mice were exposed to ozone twice a week for 6 weeks to establish COPD models, and then were intrabronchially inoculated with sterile agar beads (con-COPD) or 1.0 × 10^5^ CFU agar-entrapped P. aeruginosa (PA-COPD). Lungs were excised at one day post inoculation, and were sectioned and stained with masson trichrome to measure tissue fibrosis (**A**, upper row), and PAS to quantify glycogen [**(A)**, bottom row]. Blue areas around airways were identified as collagen deposition (indicated by black arrows), and purple areas within tracheal cavity were identified as mucus production and goblet cell hyperpasia (indicated by blue arrows). Internal scale bar = 100 μm. Quantitative real-time PCR, semiquantitative RT-PCR, and western-blot analysis of IL-23 and its receptor **(B)**, and IL-17A and its receptor **(C)** were performed using lung tissues. β-actin serves as the loading control. Data were presented as mean ± SD (*n* = 5 per group). **P < 0.01. The lungs were processed for immunofluorescent analysis **(D)**, and the sections were stained with anti-IL-17RA (red) and anti-Ly6G (green), and DAPI (blue) for nuclei. Internal scale bar = 100 μm. Two independent experiments were performed. Correlation analysis showed that there was a significantly negative correlation between the numbers of Ly6G^+^IL-17RA^+^ cells and the spirometry results **(E)**. COPD, chronic obstructive pulmonary disease; CFU, colony-forming units; IL, interleukin; FVC, forced vital capacity; FEV_50_, volume expired in the first 50 ms of fast expiration.

Having identified the increased expression of IL-23/IL-17 in BALF of *P. aeruginosa*-infected AECOPD patients, we investigated the expression of IL-23/IL-17 signaling axis in airways of COPD mouse models in response to *P. aeruginosa* infection. The COPD models were established using ozone exposure, which presented with chronic airway inflammation, mucus hypersecretion, airway remodeling and emphysema. IL-17A and IL-23 levels were higher in BALF of COPD models compared to air-control mice (**Figure S1** **).** Then COPD mouse models were transbronchially inoculated with sterile agar beads (con-COPD group) or agar-entrapped *P. aeruginosa* (PA-COPD group). We found more mucus production was observed in PA-COPD group one day post inoculation (**Figure 2A**). At both mRNA and protein levels, IL-23 and its receptor IL-23R, IL-17A and its receptor IL-17RA were significantly increased in PA-COPD lungs compared to con-COPD lungs (**Figures 2B, C**). Immunofluorescence showed that in PA-COPD lungs, more IL-17RA-positive neutrophils were seen around airway (**Figure 2D**). In addition, we found there was negative correlation between lung function and number of IL-17RA-positive neutrophils in the lungs (*r* = -0.6928, *P* = 0.0263, **Figure 2E**). The air-control mice presented with similar response when challenged with *P. aeruginosa*. However, the magnitude was less than that in COPD mice (**Figure S1**). These data suggested that genes of IL-23/IL-17-signaling pathway were over-activated by *P. aeruginosa* infection in COPD, which might play a detrimental role in the pathogenesis of airway inflammation.

### IL-17A-Signaling Aggravates Airway Dysfunction in *P. aeruginosa*-Infected COPD Mouse Models

**Figure 3:**
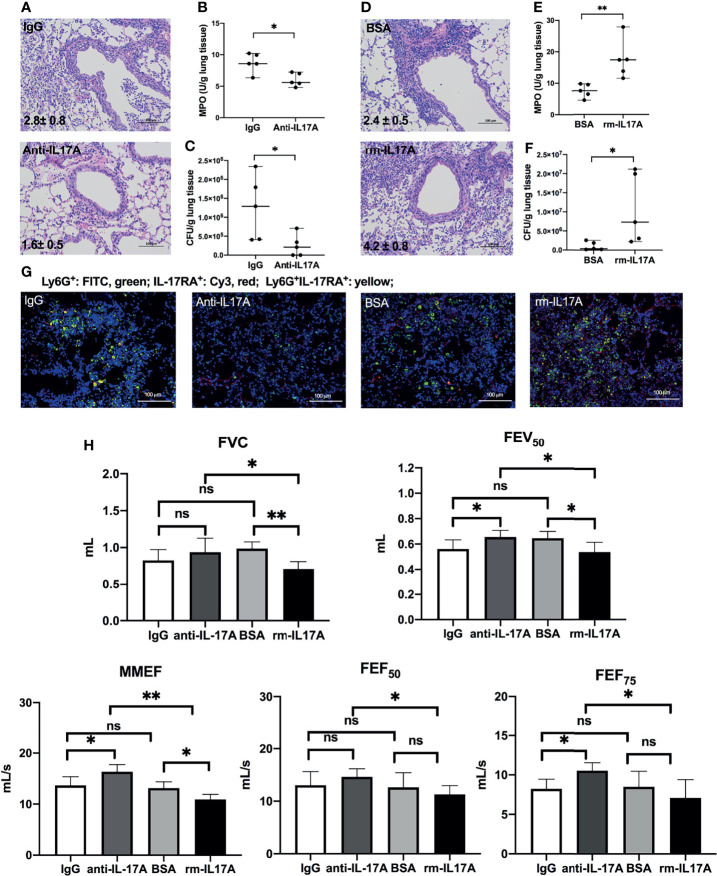
IL-17A-signaling promoted lung injury in *P. aeruginosa*-infected COPD mouse models. COPD mouse models were intraperitoneally injected with IL-17A–neutralizing antibody (2 mg/kg) or recombinant-IL-17A (1.6 mg/kg) 4 h before the inoculation with 1.0 × 10^5^ CFU *P. aeruginosa*. Mouse IgG and BSA serve as controls, respectively. Lungs were excised, sectioned and stained with hematoxylin and eosin (internal scale bar = 100 μm) at one day post inoculation **(A, D)**. The numbers within each lung microphotograph are the infiltrate scores assigned. MPO unit determination **(B, E)**, bacterial plate counting **(C, F)** were performed. The lung sections were immunofluorescently stained with anti-IL-17RA (red) and anti-Ly6G (green), and DAPI (blue) for nuclei **(G)**. Internal scale bar = 100 μm. The results of spirometry tests, including FVC, FEV_50_, MMEF, FEF_50_, and FEF_75_ were compared among different groups **(H)**. Data are presented as mean ± SD (*n* = 5). *P < 0.05, **P < 0.01, ns, non-significant. COPD, chronic obstructive pulmonary disease; MPO, myeloperoxidase; CFU, colony-forming units; BSA, bovine serum albumin; IL, interleukin; FVC, forced vital capacity; FEV_50_, volume expired in the first 50 ms of fast expiration; MMEF, maximal mid-expiratory flow; FEF_50_, forced expiratory flow at 50% FVC; FEF_75_, forced expiratory flow at 75% FVC.

To determine the role of IL-17A in the airway dysfunction induced by *P. aeruginosa* infection in COPD mouse models, we investigated the effects of IL-17A activity in PA-COPD mice by application of IL-17A–neutralizing antibody and exogenous mouse rm-IL-17A, respectively. Both IL-17A–neutralizing antibody and rm-IL-17A protein were administered 4 h prior to *P. aeruginosa* inoculation. Blockade of IL-17A resulted in reduced severity of *P. aeruginosa* airway inflammation, compared with those injected with control IgG (**Figure 3A**). The MPO activity and bacterial load assigned to anti-IL-17A mice were significantly lower than those of control IgG group (**Figures 3B, C**). We next administrated rm-IL-17A prior to *P. aeruginosa* inoculation. In contrast to blockade of IL-17A, the presence of exogenous IL-17A markedly increased the susceptibility of COPD mouse lungs to *P. aeruginosa* infection, with higher infiltrate scores (**Figure 3D**), MPO activity (**Figure 3E**) and bacterial burden (**Figure 3F**) compared with the BSA control group. Immunofluorescence analysis revealed that in contrast to IL-17A blockade, which decreased the number of infiltrated Ly-6G^+^IL-17RA^+^ cells, exogenous rm-IL-17A greatly increased the number of infiltrated Ly-6G^+^IL-17RA^+^ cells around the airways (**Figure 3G**). Both FVC and FEV_50_ were exacerbated by exogenous rm-IL-17A, however only FEV_50_ was improved by the blockade of IL-17A. On the contrary, the small airway parameters MMEF and FEF_75_ were improved by the IL-17A blockade, however only MMEF were exacerbated by rm-IL-17A (**Figure 3H**). Taken together, the results of the two complementary approaches indicated that IL-17A aggravated airway dysfunction induced by *P. aeruginosa* infection in COPD mouse models. As for air-control mice, their Ly-6G^+^IL-17RA^+^ cells were also significantly induced by IL-17A in response to *P. aeruginosa* infection, but their MPO level, bacterial burden, infiltrate scores, and lung function could not be improved by IL-17A blockade (**Figure S2** **)**.

### IL-17A Altered Immune Responses to *P. aeruginosa* Infection in COPD Mouse Models

**Figure 4:**
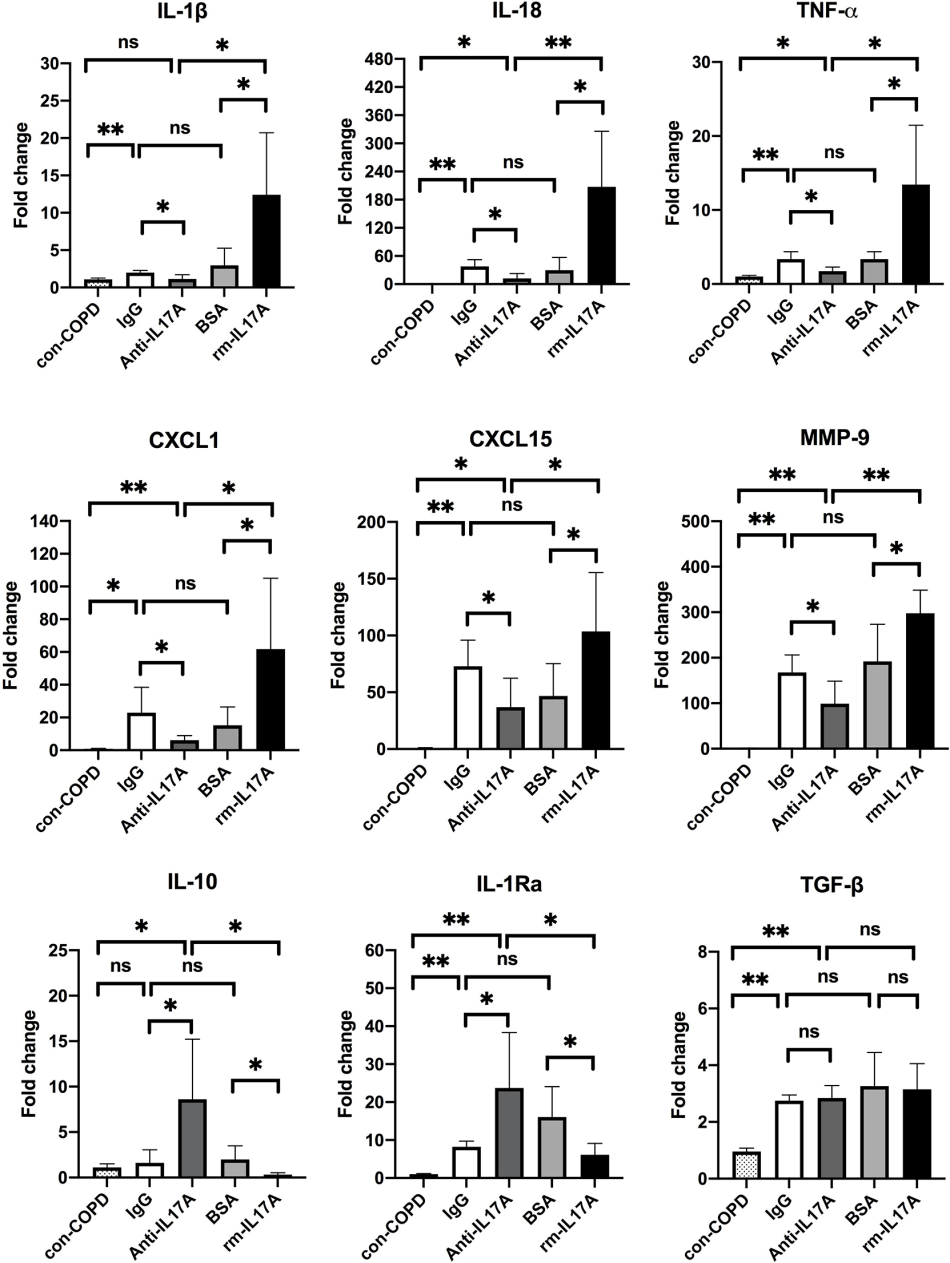
IL-17A affected transcription of inflammatory genes in responses to *P. aeruginosa* infection in COPD mouse models. Mice were intraperitoneally injected with IL-17A–neutralizing antibody (2 mg/kg) or recombinant-IL-17A (1.6 mg/kg) 4 h before the inoculation with 1.0 × 10^5^ CFU *P. aeruginosa*. Mouse IgG and BSA served as controls, respectively. COPD mouse models inoculated with sterile agar beads served as blank control (con-COPD). Lungs were excised at 24 h post inoculation and analyzed by real-time PCR. Data are presented as mean ± SD (*n* = 5 per group). *P < 0.05, **P < 0.01, ns, non-significant. COPD, chronic obstructive pulmonary disease; BSA, bovine serum albumin; IL, interleukin; CXCL, C-X-C motif chemokine ligand; MMP, matrix metalloproteinase.

**Figure 5:**
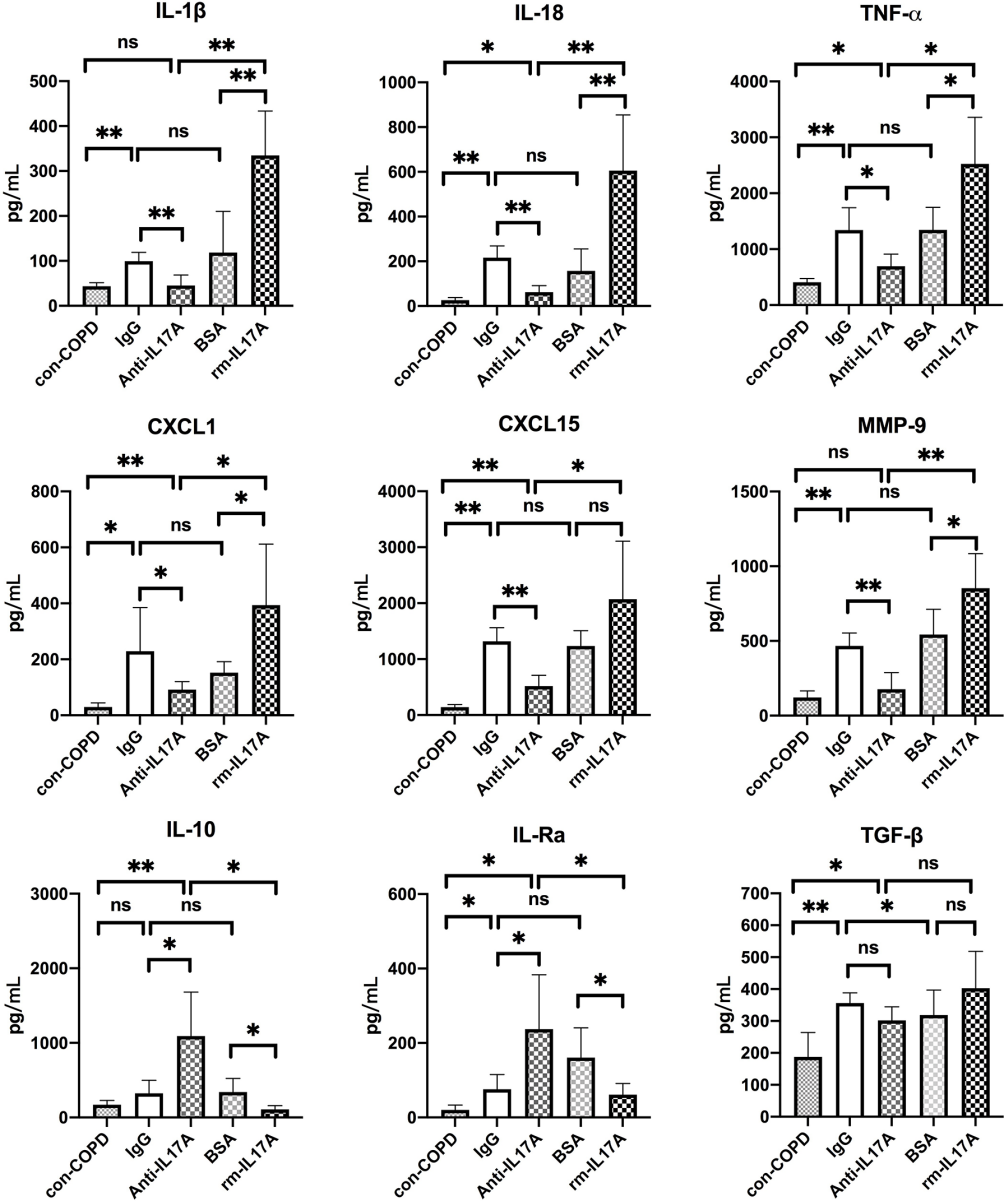
IL-17A affected protein expression of inflammatory genes in response to *P. aeruginosa* infection in COPD mouse models. Mice were intraperitoneally injected with IL-17A–neutralizing antibody (2 mg/kg) or recombinant-IL-17A (1.6 mg/kg) 4 h before the inoculation with 1.0 × 10^5^ CFU *P. aeruginosa*. Mouse IgG and BSA served as treatment controls, respectively. COPD mouse models inoculated with sterile agar beads served as blank control (con-COPD). Lungs were excised at 24 h post inoculation and analyzed by real-time PCR. Data are presented as mean ± SD (n = 5 per group). *P < 0.05, **P < 0.01, ns, non-significant. COPD, chronic obstructive pulmonary disease; BSA, bovine serum albumin; IL, interleukin; CXCL, C-X-C motif chemokine ligand; MMP, matrix metalloproteinase.

We next assessed the effects of IL-17A on the expression of several innate immune responsive genes in COPD mouse models using IL-17A–neutralizing antibody and rm–IL-17A 4 h pior to *P. aeruginosa* inoculation. We performed real-time PCR for those genes which were shown to be associated with the pathogenesis of *P. aeruginosa* infection. At one day post inoculation, IL-17A–neutralizing antibody dampened, whereas rm–IL-17A protein augmented the expression of proinflammatory genes of IL-1β, IL-18, TNF-α, CXCL1, CXCL15 and MMP-9 in response to *P. aeruginosa* infection in COPD mouse lungs. Furthermore, the expression of the anti-inflammatory gene IL-10 and IL-1Ra was augmented by IL-17A–neutralizing antibody, and dampened by rm–IL-17A protein (**Figure 4**). The protein levels of these genes were consistent with their mRNA levels (**Figure 5**). For air-control mice, only the mRNA and protein expression of IL-1β, IL-18, TNF-α, and MMP-9 were significantly affected by both IL-17A–neutralizing antibody and rm–IL-17A (**Figures S3**, **S4**).

**Figure 6:**
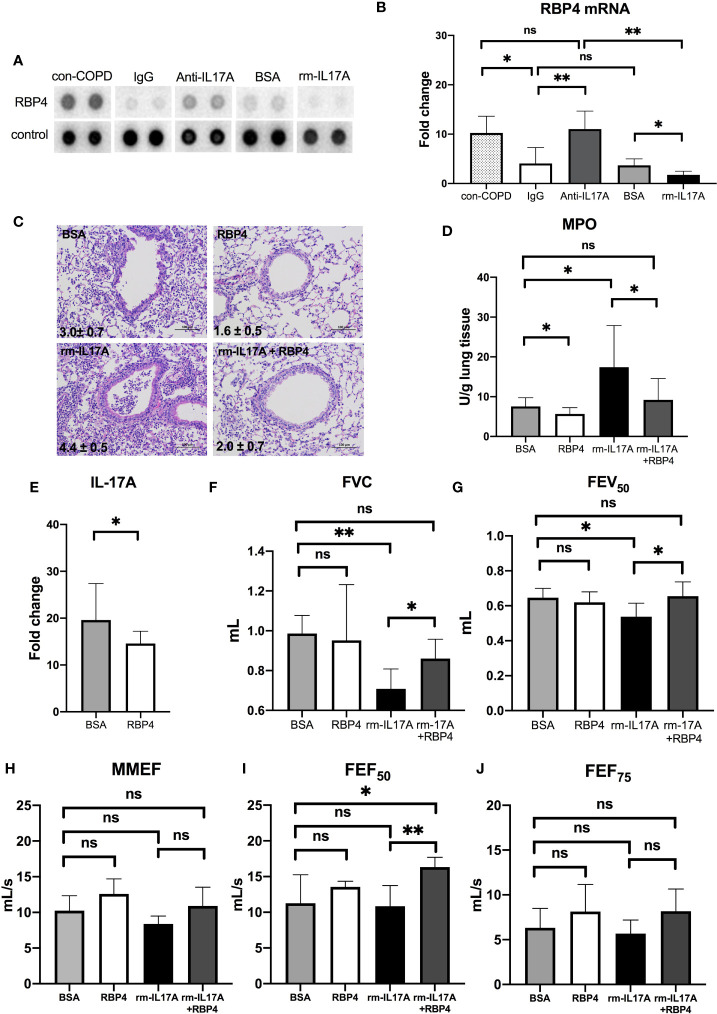
The interaction between IL-17A and RBP4 in the lungs of COPD mouse models in response to *P. aeruginosa* infection. COPD mouse models were intraperitoneally injected with IL-17A–neutralizing antibody (2mg/kg) or recombinant–IL-17A protein (1.6 mg/kg) 4 h before the inoculation with 1.0 × 10^5^ CFU *P. aeruginosa* (PA-COPD). Mouse IgG and BSA served as controls, respectively. COPD mouse models inoculated with sterile agar beads served as blank control (con-COPD). Lungs were excised at one day post inoculation. The effect of IL-17A on cytokine expression were determined using protein array analysis. Selected images for RBP4 was shown **(A)**. Realtime-PCR analysis of RBP4 were performed in lung homogenates. β-actin served as the loading control **(B)**. Data are presented as mean ± SD (*n* = 5 per group). *P < 0.05, **P < 0.01, ns = non-significant. Then COPD mouse models were intraperitoneally injected with recombinant RBP4 (5 μg/kg), recombinant–IL-17A protein (1.6 mg/kg) or IL-17A– and RBP4-recombinant protein simultaneously 4 h before the inoculation with 1.0 × 10^5^ CFU *P. aeruginosa*. Mouse BSA served as control. Mouse lungs were excised, sectioned and stained with hematoxylin and eosin (internal scale bar = 100 μm) at one day post inoculation **(C)**. The numbers within each lung microphotograph are the infiltrate scores assigned. Lungs were subjected to MPO unit determination **(D)** and real-time PCR analysis on the transcription of IL-17A **(E)**. The spirometry results, including FVC **(F)**, FEV_50_ **(G)**, MMEF **(H)**, FEF_50_ **(I)**, and FEF_75_ **(J)** were performed and compared between groups. Data were presented as mean ± SD (*n* = 5 per group). *P < 0.05, **P < 0.01. COPD, chronic obstructive pulmonary disease; BSA, bovine serum albumin; IL, interleukin; RBP4, retinol binding protein 4; MPO, myeloperoxidase; FVC, forced vital capacity; FEV_50_, volume expired in the first 50 ms of fast expiration; MMEF, maximal mid-expiratory flow; FEF_50_, forced expiratory flow at 50% FVC; FEF_75_, forced expiratory flow at 75% FVC.

We used the XL mouse cytokine array to assess the effects of IL-17A on the expression of cytokines, chemokines, and growth factors. Among 111 proteins in the array, RBP4 protein levels were abundant in con-COPD lungs and became low in PA-COPD lungs. IL-17A–neutralizing antibody increased, whereas rm–IL-17A protein further decreased, the expression of RBP4 at one day post inoculation (**Figure 6A**). Quantitative RT-PCR (qRT-PCR) was performed to confirm the results of cytokine array. We found that RBP4 transcripts were significantly down-regulated in the PA-COPD lungs in comparison to con-COPD lungs; in anti–IL-17A–treated lungs, there was a significant induction of RBP4 transcription, whereas in rm–IL-17A–treated lungs, there was a significant reduction of RBP4 transcription (**Figure 6B**).

### RBP4 Regulates IL-17A Expression, and Protect COPD Mouse Models From PA-Induced Airway Dysfunction

Having identified the regulation of RBP4 by IL-17A in PA-COPD lungs, we next investigated the role of RBP4 in the *P. aeruginosa*-induced airway dysfunction in COPD mouse models. We found RBP4 recombinant protein, which was administered 4 h prior to *P. aeruginosa* inoculation, decreased the severity of airway inflammation in PA-COPD mice, including lower infiltrate scores, and reduced MPO activity, when compared with those PA-COPD mice injected with control BSA (**Figures 6C, D**). Importantly, the RBP4 protein significantly decreased the levels of IL-17A transcripts in the lung tissues of PA-COPD mice (**Figure 6E**). When we subconjunctivally injected IL-17A– and RBP4-recombinant protein simultaneously in COPD mouse models 4 h prior to inoculation, the RBP4 protein dampened the severity of rm–IL-17A–induced airway inflammation at one day post inoculation, including a decreased infiltrate score and MPO activity compared with rm–IL-17A–only-treated PA-COPD mice as the control (**Figures 6C, D**). Although the exogenous RBP4 treatment didn’t significantly improve the lung function of PA*-*COPD mice, it alleviated the further decline of FVC and FEV_50_ caused by rm–IL-17A (**Figures 6F, G**). Among the small airway parameters, the decline of FEF_50_ was also alleviated by RBP4 (**Figures 6H–J**). Therefore, the downregulation of RBP4 by IL-17A is partially responsible for the airway dysfunction in *P. aeruginosa*-infected COPD mouse models. RBP4 treatment in PA-infected air-control mice had the similar situation (**Figure S5** **)**.

### Adjunctive Therapy of IL-17A–Neutralizing Antibody to Antibiotics Improved the Outcome of *P. aeruginosa-*Infected COPD Mouse Models

**Figure 7:**
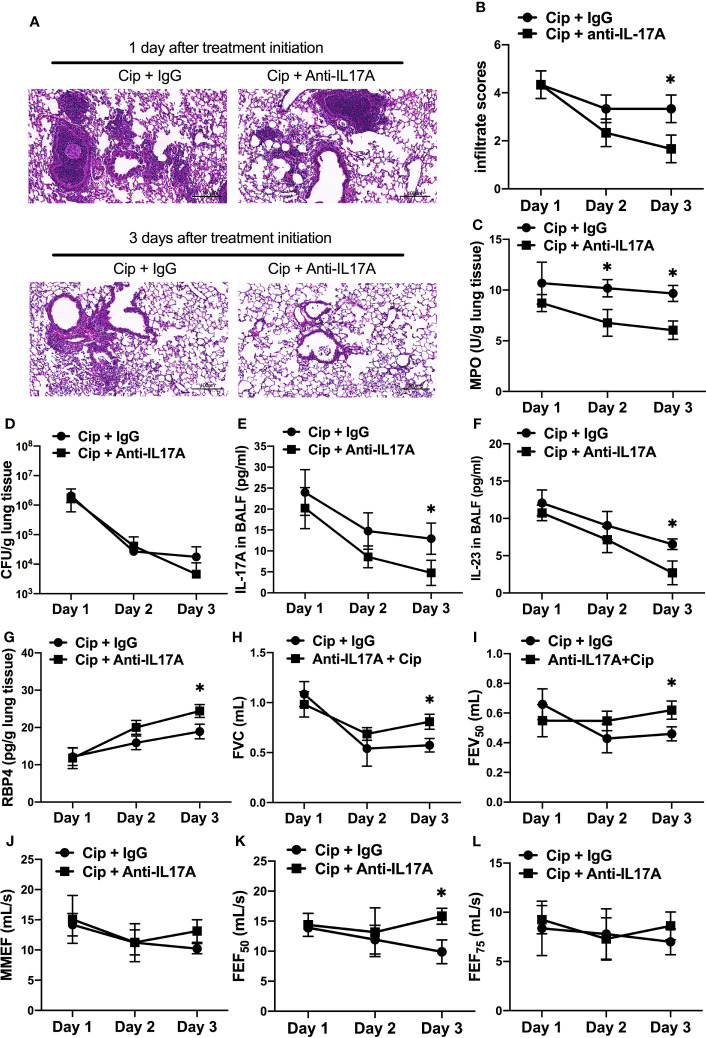
Adjunctive therapy of IL-17A–neutralizing antibody to antibiotic improved the outcome of *P. aeruginosa* infection in COPD mouse models. COPD mouse models were intrabronchially inoculated with 1.0 × 10^5^ CFU agar-entrapped *P. aeruginosa*. Oral ciprofloxacin (5 mg/kg, every 12 h) was applied concurrently with intraperitoneally administered IL-17A–neutralizing antibody (2 mg/kg, every 4 h) or IgG starting at 16 h post inoculation. Mouse lungs were excised, sectioned and stained with hematoxylin and eosin (internal scale bar = 100 μm) after treatment **(A)**, and the infiltrate scores **(B)** and MPO activity **(C)** were determined on each day. Bacterial plate counting **(D)**, protein levels of IL-17A **(E)**, IL-23 **(F)**, and RBP4 **(G)**, and spirometry tests, including FVC **(H)**, FEV_50_ **(I)**, MMEF **(J)**, FEF_50_ **(K)**, and FEF_75_ **(L)**, were performed and compared between groups treated with and without IL-17A block. Data are representative of three independent experiments, and are presented as mean ± SD (*n* = 3 per group). *P < 0.05. COPD, chronic obstructive pulmonary disease; Cip, ciprofloxacin; CFU, colony-forming units; MPO, myeloperoxidase; FVC, forced vital capacity; FEV50, volume expired in the first 50 ms of fast expiration; MMEF, maximal mid-expiratory flow; FEF_50_, forced expiratory flow at 50% FVC; FEF_75_, forced expiratory flow at 75% FVC.

To explore the potential clinical application of anti–IL-17A treatment as an adjunctive therapy in AECOPD patients **with** *P. aeruginosa* infection, we orally applied ciprofloxacin concurrently with intraperitoneally administered IL-17A–neutralizing antibody in COPD mouse models starting at 16 h post *P. aeruginosa* inoculation. The addition of anti–IL-17A antibody significantly reduced the severity of lung injury compared with control IgG on day 3 post treatment initiation (**Figure 7A**). Both the infiltrate scores and MPO levels in anti–IL-17A group was significantly decreased compared with ciprofloxacin control group (**Figures 7B, C** **)**. There was no significant difference in the bacterial clearance between two groups (**Figure 7D**). The levels of IL-23 and IL-17 were significantly decreased by combination therapy (**Figures 7E, F**), while the RBP4 level (**Figure 7G**) and lung function parameters (FVC, FEV_50_, FEF_50_) were significantly increased (**Figures 7H–L**). Hence, anti–IL-17 treatment is a potential adjunct therapy to antibiotics for treating *P. aeruginosa* infection in COPD. The combination treatment didn’t show advantage over ciprofloxacin alone in PA-infected air-control mice (**Figure S6** **)**.

## Discussion

In this study, we found IL-17 signal pathway was upregulated in COPD patients and COPD mouse models in response to *P. aeruginosa* infection, which was associated with deterioration of lung function. The blockade of IL-17A decreased, whereas rm–IL-17A exacerbated the infection-induced airway inflammation and lung dysfunction in COPD mouse models. We also found that IL-17A suppressed RBP4 expression in *P. aeruginosa*–infected mouse models, and exogenous administration of RBP4 downregulated IL-17A expression, and partially attenuated *P. aeruginosa*-induced airway dysfunction. Finally, concurrent application of IL-17A–neutralizing antibody and ciprofloxacin improved *P. aeruginosa* infection–associated inflammation and lung function in mouse models. Taken together, these results suggest that IL-17 signaling plays a pathological role in AECOPD caused by *P. aeruginosa* infection, which includes the induction of proinflammatory cytokine/chemokine and decrease in lung function. These pathological changes are partly related to the suppression of RBP4 expression.

Although IL-17A is a key factor controlling extracellular bacterial and fungal infection under normal condition, its role in AECOPD seems to be complicated (17). Previous studies reported that IL-17A was not involved in the clearance of nontypeable *Haemophilus influenzae* (NTHi) (31), and its response to *Streptococcus Pneumoniae* (32) was defective during COPD exacerbations. Those studies suggested the protective role of IL-17A against bacteria infection was limited in AECOPD. In this study, we found in the mouse models of COPD, despite decreased neutrophil recruitment after treatment with IL-17A–neutralizing antibody, no increased bacterial burden was observed in the lungs, suggesting IL-17A was not critical in the clearance of *P. aeruginosa* during AECOPD. Instead, we found IL-17A induced severe inflammation in the airways and caused lung function decline in mouse models. Our data supported the impaired balance between the role of antibacterial and hyperinflammatory activities for IL-17A in AECOPD, which could result in the bacterial colonisation and persistent neutrophilic inflammation in the airways.

IL-23 is an important mediator of tissue inflammation, which induces the differentiation of naive CD4^+^ T cells into highly pathogenic helper T cells (Th17) that produce IL-17A, IL-17F, IL-6, and TNF-α, but not IFN-γ and IL-4 (33). In addition, IL-23 produced by residential dendritic cells is likely to be an initial step in the inflammatory cascade that drives the infiltration of innate defense cells such as neutrophils, NK cells, and innate lymphoid cells, most of which are capable of secreting IL-17A (34). Our results showed that IL-17A and IL-23 concentrations were significantly increased in BALF of AECOPD patients with *P. aeruginosa* infection, and there was a notable positive correlation between the two cytokines. The mouse models also showed increased expressions of IL-23/17 axis–signaling molecules by *P. aeruginosa* infection, including IL-23, IL-23R, IL-17A, and IL-17RA. Notably, the infiltrated neutrophils, which were IL-17RA positive, were increased around airways. These data indicated the involvement of IL-23/IL-17 axis in the neutrophilic airway inflammation by *P. aeruginosa* infection in AECOPD.

To investigate the mechanism by which IL-17A drives airway inflammatory response to *P. aeruginosa* infection in COPD, we assessed the effects of IL-17A on the expression of genes known to be involved in the airway immune defense against microbial infection. We found that IL-17A–neutralizing antibody dampened, whereas rm–IL-17A protein augmented the expression of proinflammatory genes of IL-1β, IL-18, TNF-α, CXCL1, CXCL15 and MMP-9 in the lung tissues one day after *P. aeruginosa* inoculation. In contrast, the expression of the anti-inflammatory gene IL-10 and IL-1Ra was augmented by IL-17A–neutralizing antibody, and dampened by rm–IL-17A. IL-1β and IL-18 are members of the IL-1 family, and both induces T cells to produce IL-17 and promote autoimmune responses to specific antigens (35). CXCL1 and CXCL15 are potent neutrophil chemoattractants, which can be released by airway epithelial cells in the response of IL-17 signaling against *P. aeruginosa* (36, 37), whereas IL-10 and IL-1Ra are anti-inflammatory cytokines that counteracts LPS in recruiting neutrophils (38, 39). TNF-α is known to trigger cell activation, migration, or proliferation against pathogens, and is involved in the pathogenesis of inflammatory and autoimmune diseases. TNF-α can induce the release of cytokines IL-6, IL-1β, and IL-8, which can be enhanced by IL-17 (40). MMP-9 is produced by a variety of cells including epithelial cells, fibroblasts, dendritic cells, macrophages, and granulocyteshas in response to *P. aeruginosa* infection, and has the capacity to degrade both elastin and partially hydrolyzed collagen resulting in lung structure injury (41). Our data supported that IL-17 signaling can increase the expression of proinflammatory cytokines and decrease the expression of anti-inflammatory cytokines in *P. aeruginosa*-infected COPD mouse models, which suggested that IL-17 signaling blockade is a potential approach to resolute infection-associated inflammation in AECOPD.

As we are interested in exploring the mechanisms underlying IL-17A’s influence on *P. aeruginosa* infection in AECOPD, a cytokine protein array was used, which showed that the protein levels of RBP4 were most dramatically down-regulated in the lungs of COPD mouse models in response to *P. aeruginosa* infection. Furthermore, IL-17A neutralizing promoted, and exogenous IL-17A suppressed, the transcription and expression of RBP4 in the lung tissues. In the functional study, exogenous RBP4 attenuated the severity of IL-17A-induced neutrophilic airway inflammation. RBP4 is synthesized in the liver and adipose tissue, and is the circulating transporter for vitamin A. Previous reports indicated that RBP4 levels in patients with AECOPD were significantly lower than those in stable COPD and healthy subjects (42, 43). Serum RBP4 levels were associated with nutritional status, which is related to respiratory impairment and systemic inflammation in patients with AECOPD (44). Malnutrition increases the incidence of complications and mortality, and can be identified as a risk factor associated with short-term mortality of elderly AECOPD patients. Our data showed that IL-17A signaling could influence the production of RBP4, which to our knowledge is the first report to link RBP4 expression to IL-17 signaling in AECOPD that caused by *P. aeruginosa* infection. It’s notable that the exogenous RBP4 could downregulate IL-17A transcription, suggesting a negative feedback response of RBP4 expression to IL-17A signaling. Our data supported that AECOPD patients with *P. aeruginosa* infection might benefit from vitamin A supplementation during IL-17-induced inflammatory injury. The link between IL-17 and RBP4 in *P. aeruginosa-*infected AECOPD still needs further investigation.

At last, we tested therapeutic potential of IL-17 neutralization on controlling infection-induced airway inflammation in combination with the treatment of oral antibiotics in *P. aeruginosa-*infected AECOPD. Although ciprofloxacin is one of the most active drugs against *P. aeruginosa* among the quinolones (45), inflammation may remain due to the virulence factors, such as LPS, released by the dissolved pathogen (46). Our data showed that the combination treatment of ciprofloxacin and IL-17A–neutralizing antibody reduced airway inflammation associated with *P. aeruginosa* infection as assessed by infiltrate scores and MPO examination. Notably, IL-17A neutralizing could benefit the improvement of lung function in COPD mice after *P. aeruginisa* infection. These data suggested the value of applying concurrent therapy of IL-17A–neutralizing antibody and antibiotics in the treatment of *P. aeruginisa*-infected AECOPD.

Our study focused on IL-17-medated cytokine alternation and its impact on lung function and histopathology in AECOPD with *P. aeruginosa* infection. Limitation of this study included lack of molecular mechanisms on celluar immunity and cross-regulation between IL-17A and RBP4 during AECOPD. Further investigations are needed to support these results of our study.

In conclusion, our study indicated that IL-17 signaling pathway aggravated *P. aeruginosa*-induced airway dysfunction in AECOPD, which is partly related to the downregulation of RBP4 expression. These data supported the therapeutic value of targeting IL-17A in the treatment of *P. aeruginosa* infection in AECOPD.

## Data Availability Statement

The original contributions presented in the study are included in the article/**Supplementary Material**. Further inquiries can be directed to the corresponding author.

## Ethics Statement

The studies involving human participants were reviewed and approved by the Ethics Committee of Shanghai General Hospital. The patients/participants provided their written informed consent to participate in this study. The animal study was reviewed and approved by the institutional animal care and use committee of Shanghai Jiao Tong University.

## Authors Contributions

FD, LH, and MZ conceived of and designed the entire study. XT, RT, and CL contributed to data collection and statistical analyses. YX, LH, and QF performed the experiments. FD and LH wrote the manuscript, supervised by MZ. All authors critically reviewed and approved the final version. All authors agreed to be accountable for all aspects of the work in ensuring that questions related to the accuracy or integrity of any part of the work are appropriately investigated and resolved.

## Funding

This work was supported by the National Natural Science Foundation of China (Grant No. 81970006 and Grant No. 81873402); Project of Science and Technology Commission of Shanghai Municipality (Grant No. 20ZR1444300, Grant No. 20Y11902400 and Grant No. 20Z11900903); Appropriate Technique Application Program of Shanghai Municipal Health System (Grant No. 2019SY042); Three-year Action Plan of Shanghai Shenkang Hospital Development Center (Grant No. SHDC2020CR5010).

## Conflict of Interest

The authors declare that the research was conducted in the absence of any commercial or financial relationships that could be construed as a potential conflict of interest.

## Publisher’s Note

All claims expressed in this article are solely those of the authors and do not necessarily represent those of their affiliated organizations, or those of the publisher, the editors and the reviewers. Any product that may be evaluated in this article, or claim that may be made by its manufacturer, is not guaranteed or endorsed by the publisher.

## Supplementary Material

Supplementary Figure 1 IL-23/IL-17 axis signaling between the lungs of COPD mouse models and air-control mice in response to *P. aeruginosa* infection. C57BL/6 mice were exposed to ozone twice a week for 6 weeks to establish COPD models, and then were intrabronchially inoculated with sterile agar beads (con-COPD) or 1.0 × 10^5^ CFU agar-entrapped *P. aeruginosa* (PA-COPD). Mice exposed to air were used as controls and also inoculated with sterile agar beads (con-air) or 1.0 × 10^5^ CFU agar-entrapped *P. aeruginosa* (PA-air). Mice were euthanized at one day post inoculation. Quantitative real-time PCR and ELISA analysis of IL-23 **(A, B)**, and IL-17A **(C, D)** were performed using lung tissues or BALF. Data were presented as mean ± SD (*n* = 5 per group). **P* < 0.01, ***P* < 0.01. The lungs were processed for immunofluorescent analysis **(E)**, and correlation analysis showed that there was a significantly negative correlation between the numbers of Ly6G^+^IL-17RA^+^ cells and spirometry results in the study population**(F)**. COPD, chronic obstructive pulmonary disease; CFU, colony-forming units; IL, interleukin; FVC, forced vital capacity; FEV_50_, volume expired in the first 50 ms of fast expiration.

Supplementary Figure 2 The effect of IL-17A-signaling on lung injury in *P. aeruginosa*-infected air-control mice. C57BL/6 control mice that exposed to air were intrabronchially inoculated with sterile agar beads (con-air) or 1.0 × 10^5^ CFU agar-entrapped *P. aeruginosa* (PA-air). Mice were intraperitoneally injected with IL-17A–neutralizing antibody (2 mg/kg) or recombinant-IL-17A (1.6 mg/kg) 4 h before the inoculation with 1.0 × 10^5^ CFU *P. aeruginosa*. Mouse IgG and BSA serve as treatment controls, respectively. Lungs were excised, sectioned and stained with hematoxylin and eosin at one day post inoculation. MPO unit determination **(A)**, bacterial plate counting **(B)** and infiltrate scoring **(C)** were performed. The lung sections were analysed immunofluorescently for the expression of Ly6G^+^IL-17RA^+^ cells **(D)**. The results of spirometry tests, including FVC **(E)**, FEV_50_ **(F)**, MMEF **(G)**, FEF_50_ **(H)**, and FEF_75_ **(I)** were compared among different groups. Data are presented as mean ± SD (*n* = 5). **P* < 0.05, ***P* < 0.01. COPD, chronic obstructive pulmonary disease; MPO, myeloperoxidase; CFU, colony-forming units; BSA, bovine serum albumin; FVC, forced vital capacity; FEV_50_, volume expired in the first 50 ms of fast expiration; MMEF, maximal mid-expiratory flow; FEF_50_, forced expiratory flow at 50% FVC; FEF_75_, forced expiratory flow at 75% FVC.

Supplementary Figure 3 The effect of IL-17A on inflammatory gene transcription in response to *P. aeruginosa* infection by air-control mice. C57BL/6 control mice that exposed to air were intraperitoneally injected with IL-17A–neutralizing antibody (2 mg/kg) or recombinant-IL-17A (1.6 mg/kg) 4 h before the inoculation with 1.0 × 10^5^ CFU *P. aeruginosa* (PA-air). Mouse IgG and BSA served as treatment controls, respectively. Mice inoculated with sterile agar beads served as blank control (con-air). Lungs were excised at 24 h post inoculation and analyzed by real-time PCR. Data are presented as mean ± SD (n = 5 per group). **P* < 0.05, ***P* < 0.01. COPD, chronic obstructive pulmonary disease; BSA, bovine serum albumin; IL, interleukin; CXCL, C-X-C motif chemokine ligand; MMP, matrix metalloproteinase.

Supplementary Figure 4 The effect of IL-17A on inflammatory factor expression in response to *P. aeruginosa* infection by air-control mice. C57BL/6 control mice that exposed to air were intraperitoneally injected with IL-17A–neutralizing antibody (2 mg/kg) or recombinant-IL-17A (1.6 mg/kg) 4 h before the inoculation with 1.0 × 10^5^ CFU *P. aeruginosa* (PA-air). Mouse IgG and BSA served as treatment controls, respectively. Mice inoculated with sterile agar beads served as blank control (con-air). Lungs were excised at 24 h post inoculation and analyzed by real-time PCR. Data are presented as mean ± SD (n = 5 per group). **P* < 0.05, ***P* < 0.01. COPD, chronic obstructive pulmonary disease; BSA, bovine serum albumin; IL, interleukin; CXCL, C-X-C motif chemokine ligand; MMP, matrix metalloproteinase.

Supplementary Figure 5 The interaction between IL-17A and RBP4 in the lungs of air-control mice in response to *P. aeruginosa* infection. C57BL/6 control mice that exposed to air were intraperitoneally injected with IL-17A–neutralizing antibody (2mg/kg) or recombinant–IL-17A protein (1.6 mg/kg) 4 h before the inoculation with 1.0 × 10^5^ CFU *P. aeruginosa* (PA-air). Mouse IgG and BSA served as treatment controls, respectively. Mice inoculated with sterile agar beads served as blank control (con-air). Realtime-PCR and ELISA analysis of RBP4 were performed in lung homogenates **(A, B)**. Data are presented as mean ± SD (*n* = 5 per group). **P* < 0.05, ***P* < 0.01. Then air-control mice were intraperitoneally injected with recombinant RBP4 (5 μg/kg), recombinant–IL-17A protein (1.6 mg/kg) or IL-17A– and RBP4-recombinant protein simultaneously 4 h before the inoculation with 1.0 × 10^5^ CFU *P. aeruginosa*. Mouse BSA served as control. Lungs were excised and subjected to MPO unit determination **(C)**. The spirometry results, including FVC (D), FEV_50_ **(E)**, MMEF **(F)**, FEF_50_ **(G)**, and FEF_75_ **(H)** were performed and compared between groups. Data were presented as mean ± SD (*n* = 5 per group). **P* < 0.05, ***P* < 0.01. COPD, chronic obstructive pulmonary disease; BSA, bovine serum albumin; IL, interleukin; RBP4, retinol binding protein 4; MPO, myeloperoxidase; FVC, forced vital capacity; FEV_50_, volume expired in the first 50 ms of fast expiration; MMEF, maximal mid-expiratory flow; FEF_50_, forced expiratory flow at 50% FVC; FEF_75_, forced expiratory flow at 75% FVC.

Supplementary Figure 6 Combination therapy of IL-17A–neutralizing antibody and antibiotic in *P. aeruginosa*-infected air-control mice. C57BL/6 control mice that exposed to air were intrabronchially inoculated with 1.0 × 10^5^ CFU agar-entrapped *P. aeruginosa*. Oral ciprofloxacin (5 mg/kg, every 12 h) was applied concurrently with intraperitoneally administered IL-17A–neutralizing antibody (2 mg/kg, every 4 h) or IgG starting at 16 h post inoculation. Mouse lungs were excised, sectioned and stained with hematoxylin and eosin after treatment. The infiltrate scores **(A)**, MPO activity **(B)**, bacterial plate counting **(C)**, Ly6G^+^IL-17RA^+^ cell numbers **(D)**, protein expression of IL-17 **(E)** and IL-23 **(F)**, and RBP4 **(G)**, and spirometry tests, including FVC **(H)**, FEV_50_ **(I)**, MMEF **(J)**, FEF_50_ **(K)**, and FEF_75_ **(L)**, were performed and compared between groups treated with and without IL-17A block. Data are representative of three independent experiments, and are presented as mean ± SD (*n* = 3 per group). **P* < 0.05, ***P* < 0.01. COPD, chronic obstructive pulmonary disease; Cip, ciprofloxacin; CFU, colony-forming units; MPO, myeloperoxidase; FVC, forced vital capacity; FEV50, volume expired in the first 50 ms of fast expiration; MMEF, maximal mid-expiratory flow; FEF_50_, forced expiratory flow at 50% FVC; FEF_75_, forced expiratory flow at 75% FVC.
