# Supplementary figures and images for: IL-17 Aggravates Pseudomonas aeruginosa Airway Infection in Acute Exacerbations of Chronic Obstructive Pulmonary Disease
Source: Front Immunol. 2022 Jan 13;12:811803. doi: 10.3389/fimmu.2021.811803 (PMC8792752; doi:10.3389/fimmu.2021.811803)

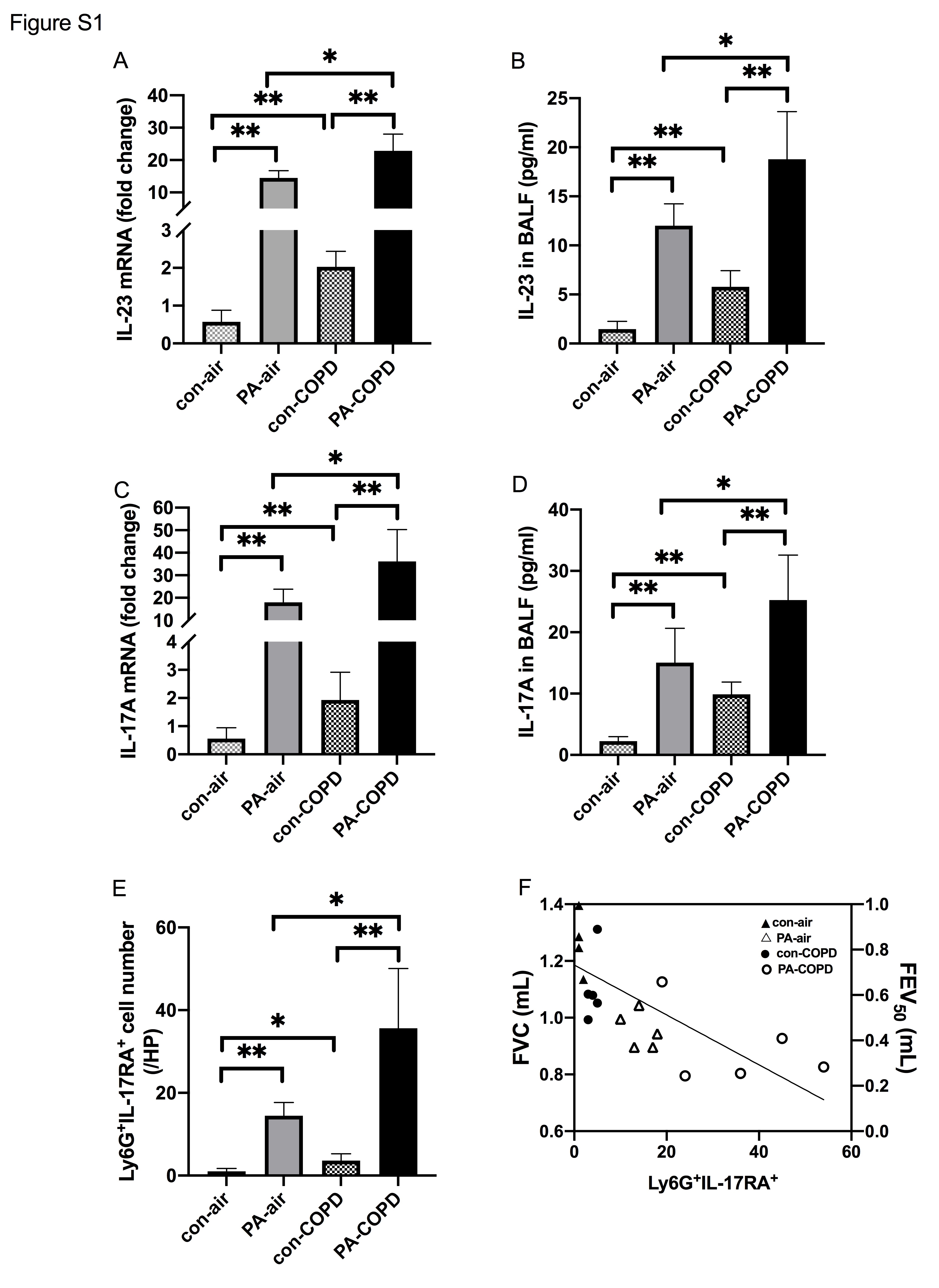

Supplement: Supplementary Figure 1 — IL-23/IL-17 axis signaling between the lungs of COPD mouse models and air-control mice in response to P. aeruginosa infection. C57BL/6 mice were exposed to ozone twice a week for 6 weeks to establish COPD models, and then were intrabronchially inoculated with sterile agar beads (con-COPD) or 1.0 × 10^5^ CFU agar-entrapped P. aeruginosa (PA-COPD). Mice exposed to air were used as controls and also inoculated with sterile agar beads (con-air) or 1.0 × 10^5^ CFU agar-entrapped P. aeruginosa (PA-air). Mice were euthanized at one day post inoculation. Quantitative real-time PCR and ELISA analysis of IL-23 (A, B), and IL-17A (C, D) were performed using lung tissues or BALF. Data were presented as mean ± SD (n = 5 per group). *P < 0.01, **P < 0.01. The lungs were processed for immunofluorescent analysis (E), and correlation analysis showed that there was a significantly negative correlation between the numbers of Ly6G+IL-17RA+ cells and spirometry results in the study population(F). COPD, chronic obstructive pulmonary disease; CFU, colony-forming units; IL, interleukin; FVC, forced vital capacity; FEV50, volume expired in the first 50 ms of fast expiration. [file Image_1.jpeg]

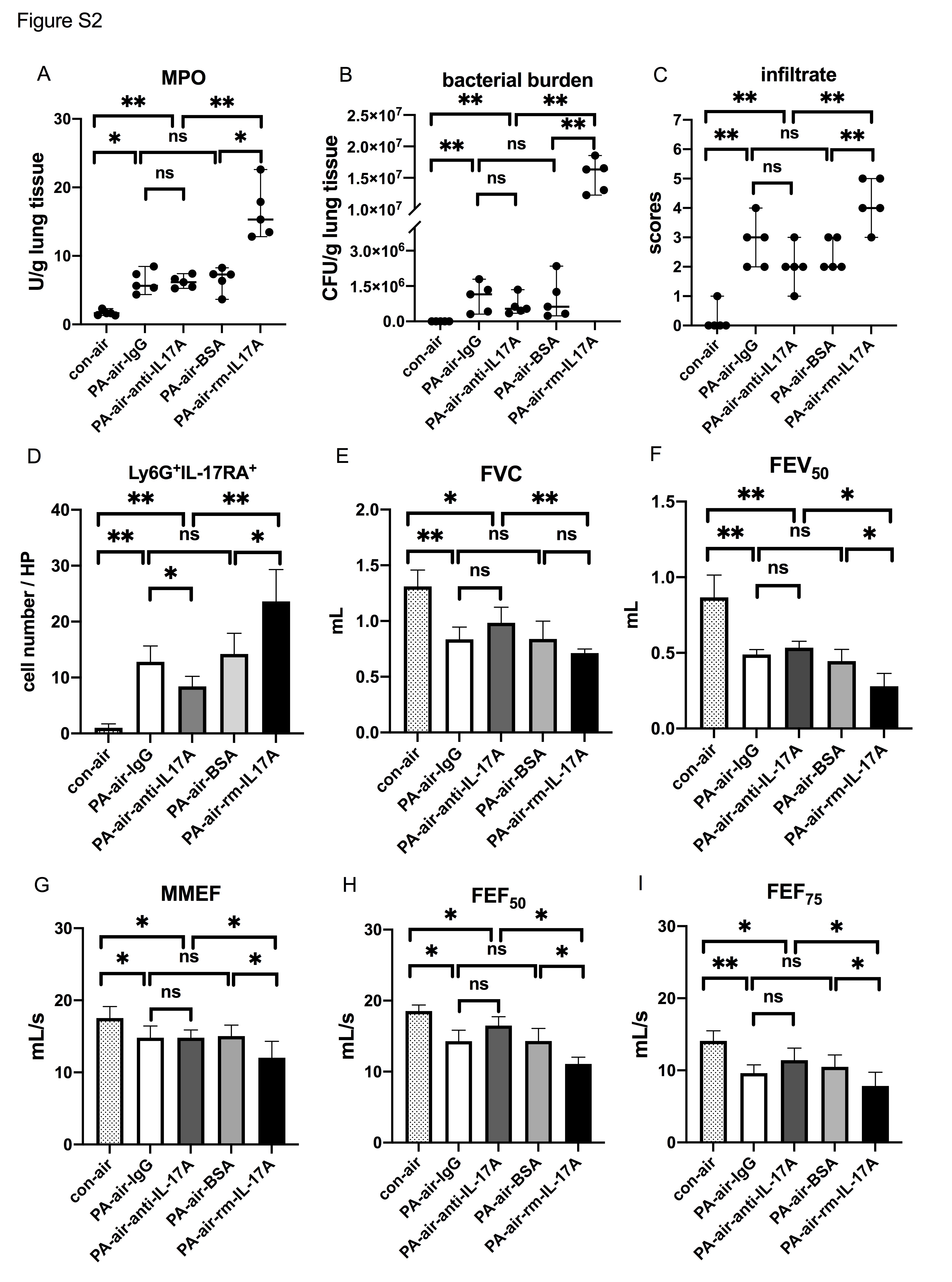

Supplement: Supplementary Figure 2 — The effect of IL-17A-signaling on lung injury in P. aeruginosa-infected air-control mice. C57BL/6 control mice that exposed to air were intrabronchially inoculated with sterile agar beads (con-air) or 1.0 × 10^5^ CFU agar-entrapped P. aeruginosa (PA-air). Mice were intraperitoneally injected with IL-17A–neutralizing antibody (2 mg/kg) or recombinant-IL-17A (1.6 mg/kg) 4 h before the inoculation with 1.0 × 10^5^ CFU P. aeruginosa. Mouse IgG and BSA serve as treatment controls, respectively. Lungs were excised, sectioned and stained with hematoxylin and eosin at one day post inoculation. MPO unit determination (A), bacterial plate counting (B) and infiltrate scoring (C) were performed. The lung sections were analysed immunofluorescently for the expression of Ly6G+IL-17RA+ cells (D). The results of spirometry tests, including FVC (E), FEV50 (F), MMEF (G), FEF50 (H), and FEF75 (I) were compared among different groups. Data are presented as mean ± SD (n = 5). *P < 0.05, **P < 0.01. COPD, chronic obstructive pulmonary disease; MPO, myeloperoxidase; CFU, colony-forming units; BSA, bovine serum albumin; FVC, forced vital capacity; FEV50, volume expired in the first 50 ms of fast expiration; MMEF, maximal mid-expiratory flow; FEF50, forced expiratory flow at 50% FVC; FEF75, forced expiratory flow at 75% FVC. [file Image_2.jpeg]

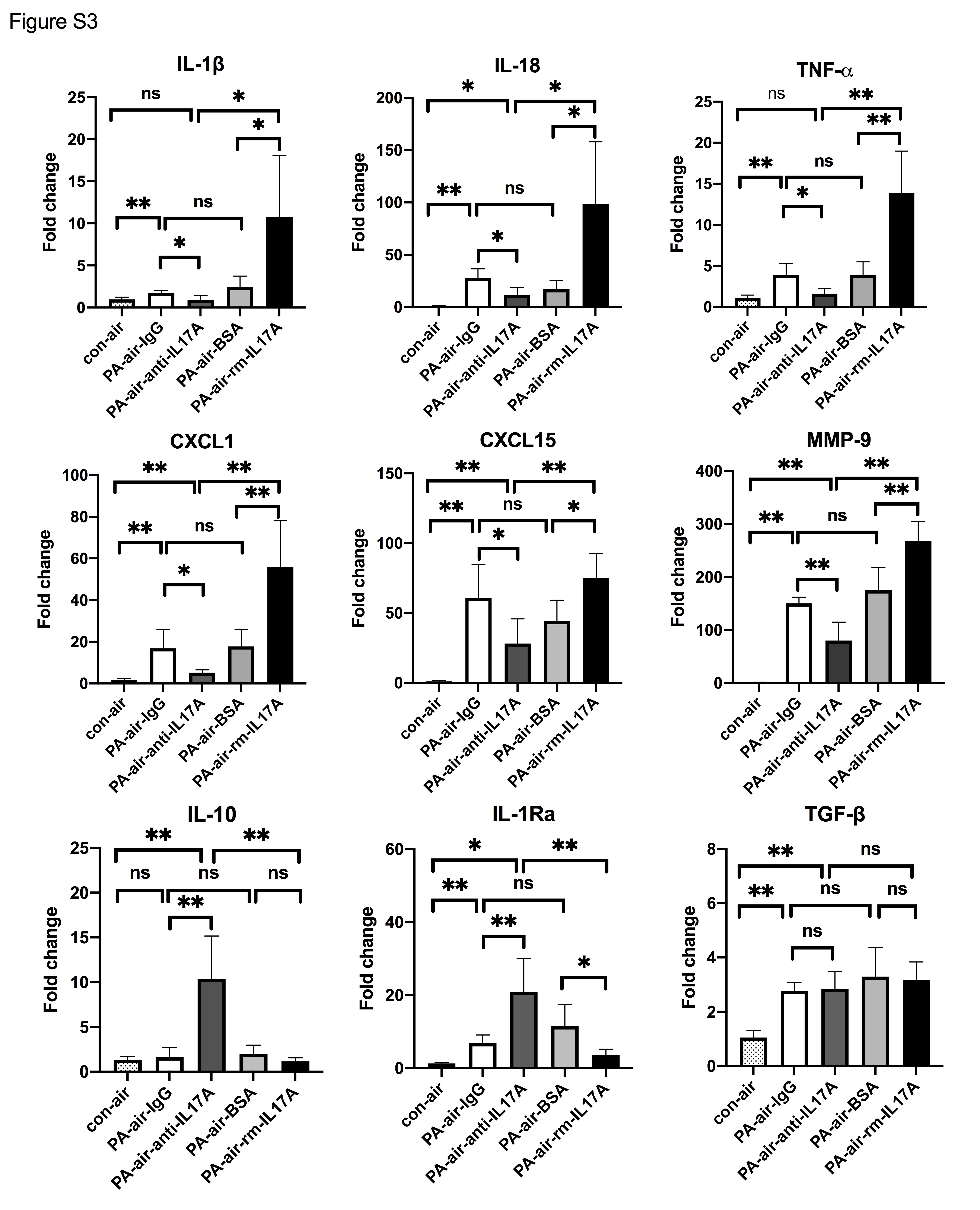

Supplement: Supplementary Figure 3 — The effect of IL-17A on inflammatory gene transcription in response to P. aeruginosa infection by air-control mice. C57BL/6 control mice that exposed to air were intraperitoneally injected with IL-17A–neutralizing antibody (2 mg/kg) or recombinant-IL-17A (1.6 mg/kg) 4 h before the inoculation with 1.0 × 10^5^ CFU P. aeruginosa (PA-air). Mouse IgG and BSA served as treatment controls, respectively. Mice inoculated with sterile agar beads served as blank control (con-air). Lungs were excised at 24 h post inoculation and analyzed by real-time PCR. Data are presented as mean ± SD (n = 5 per group). *P < 0.05, **P < 0.01. COPD, chronic obstructive pulmonary disease; BSA, bovine serum albumin; IL, interleukin; CXCL, C-X-C motif chemokine ligand; MMP, matrix metalloproteinase. [file Image_3.jpeg]

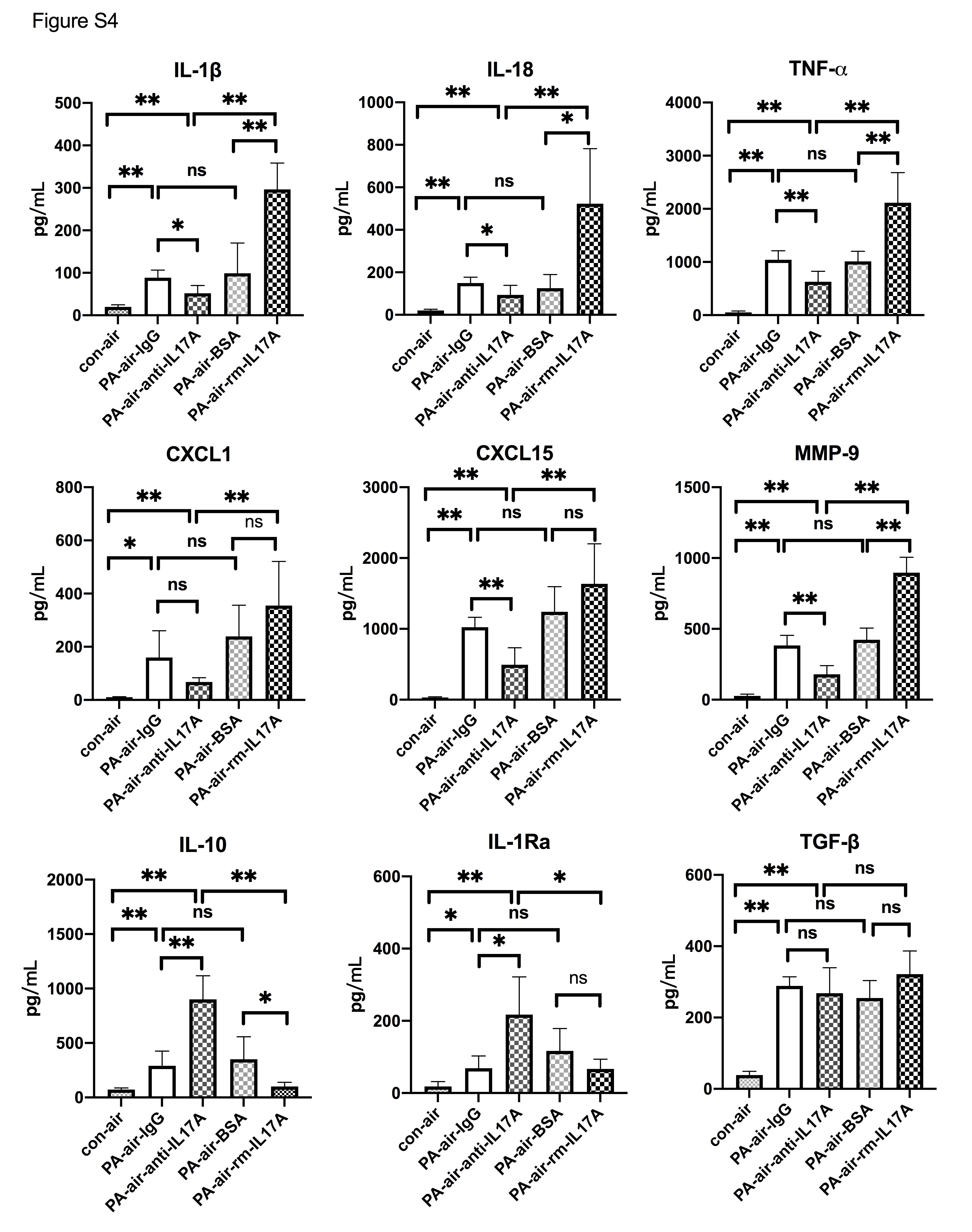

Supplement: Supplementary Figure 4 — The effect of IL-17A on inflammatory factor expression in response to P. aeruginosa infection by air-control mice. C57BL/6 control mice that exposed to air were intraperitoneally injected with IL-17A–neutralizing antibody (2 mg/kg) or recombinant-IL-17A (1.6 mg/kg) 4 h before the inoculation with 1.0 × 10^5^ CFU P. aeruginosa (PA-air). Mouse IgG and BSA served as treatment controls, respectively. Mice inoculated with sterile agar beads served as blank control (con-air). Lungs were excised at 24 h post inoculation and analyzed by real-time PCR. Data are presented as mean ± SD (n = 5 per group). *P < 0.05, **P < 0.01. COPD, chronic obstructive pulmonary disease; BSA, bovine serum albumin; IL, interleukin; CXCL, C-X-C motif chemokine ligand; MMP, matrix metalloproteinase. [file Image_4.jpeg]

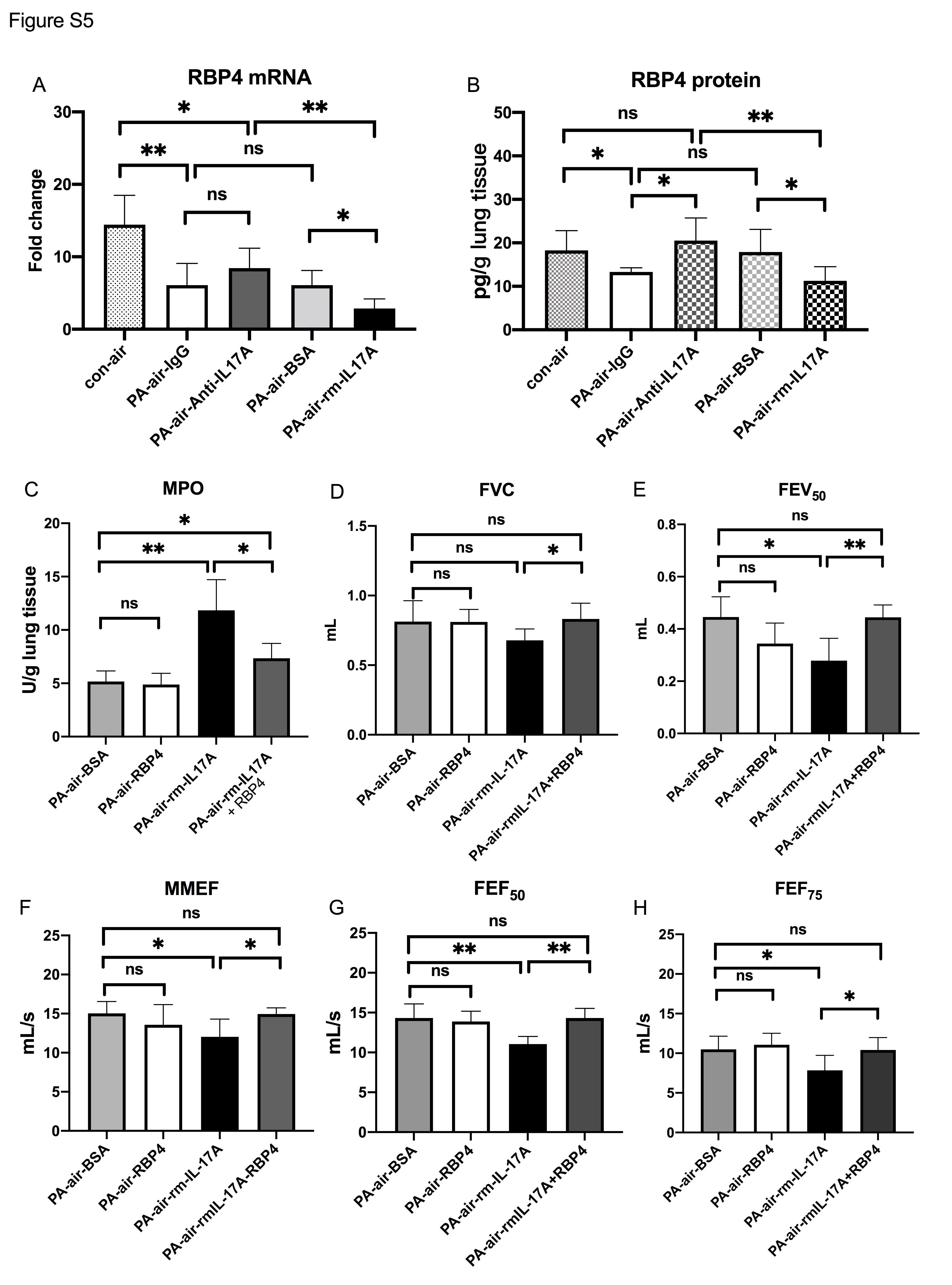

Supplement: Supplementary Figure 5 — The interaction between IL-17A and RBP4 in the lungs of air-control mice in response to P. aeruginosa infection. C57BL/6 control mice that exposed to air were intraperitoneally injected with IL-17A–neutralizing antibody (2mg/kg) or recombinant–IL-17A protein (1.6 mg/kg) 4 h before the inoculation with 1.0 × 10^5^ CFU P. aeruginosa (PA-air). Mouse IgG and BSA served as treatment controls, respectively. Mice inoculated with sterile agar beads served as blank control (con-air). Realtime-PCR and ELISA analysis of RBP4 were performed in lung homogenates (A, B). Data are presented as mean ± SD (n = 5 per group). *P < 0.05, **P < 0.01. Then air-control mice were intraperitoneally injected with recombinant RBP4 (5 μg/kg), recombinant–IL-17A protein (1.6 mg/kg) or IL-17A– and RBP4-recombinant protein simultaneously 4 h before the inoculation with 1.0 × 10^5^ CFU P. aeruginosa. Mouse BSA served as control. Lungs were excised and subjected to MPO unit determination (C). The spirometry results, including FVC (D), FEV50 (E), MMEF (F), FEF50 (G), and FEF75 (H) were performed and compared between groups. Data were presented as mean ± SD (n = 5 per group). *P < 0.05, **P < 0.01. COPD, chronic obstructive pulmonary disease; BSA, bovine serum albumin; IL, interleukin; RBP4, retinol binding protein 4; MPO, myeloperoxidase; FVC, forced vital capacity; FEV50, volume expired in the first 50 ms of fast expiration; MMEF, maximal mid-expiratory flow; FEF50, forced expiratory flow at 50% FVC; FEF75, forced expiratory flow at 75% FVC. [file Image_5.jpeg]

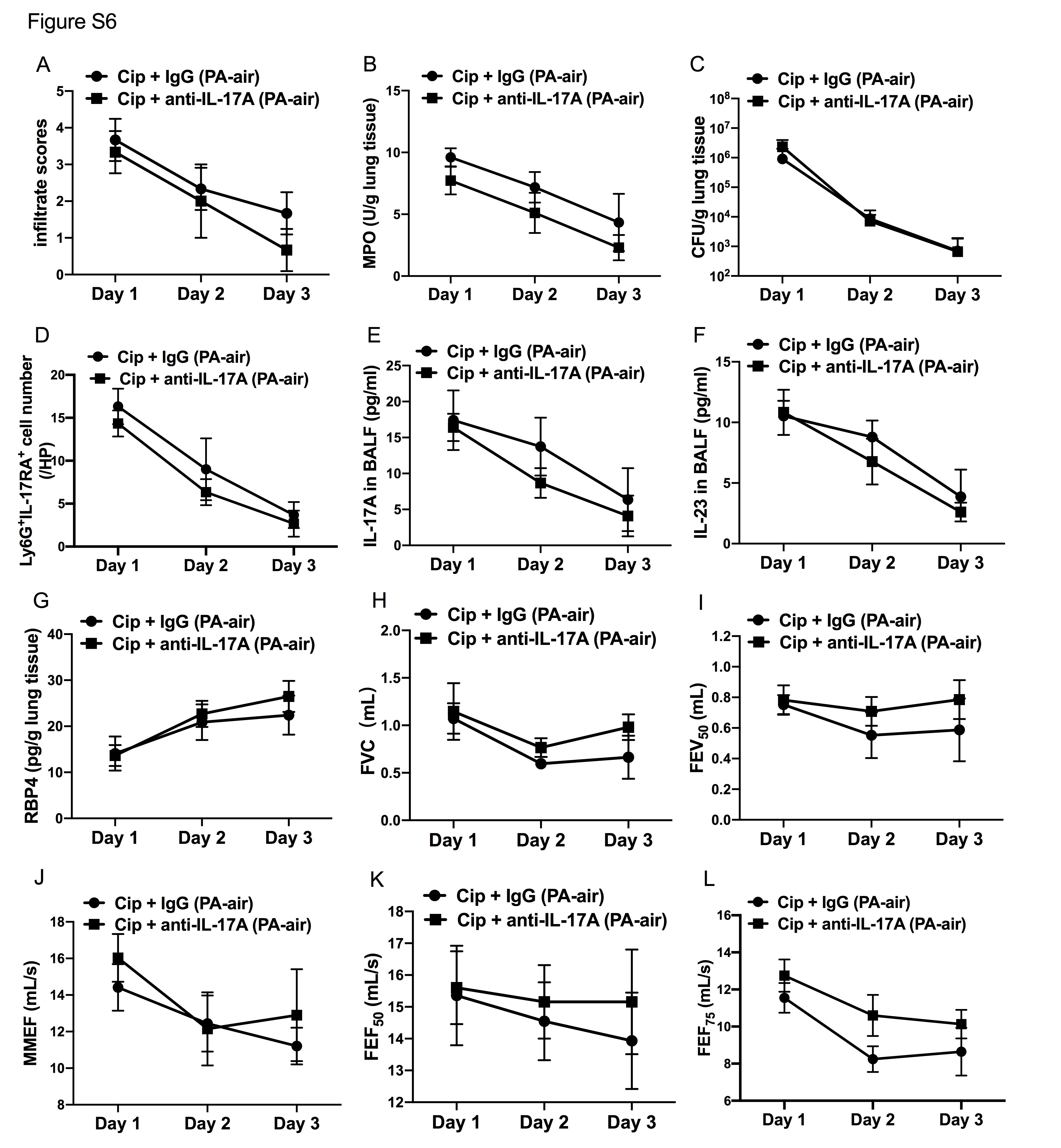

Supplement: Supplementary Figure 6 — Combination therapy of IL-17A–neutralizing antibody and antibiotic in P. aeruginosa-infected air-control mice. C57BL/6 control mice that exposed to air were intrabronchially inoculated with 1.0 × 10^5^ CFU agar-entrapped P. aeruginosa. Oral ciprofloxacin (5 mg/kg, every 12 h) was applied concurrently with intraperitoneally administered IL-17A–neutralizing antibody (2 mg/kg, every 4 h) or IgG starting at 16 h post inoculation. Mouse lungs were excised, sectioned and stained with hematoxylin and eosin after treatment. The infiltrate scores (A), MPO activity (B), bacterial plate counting (C), Ly6G+IL-17RA+ cell numbers (D), protein expression of IL-17 (E) and IL-23 (F), and RBP4 (G), and spirometry tests, including FVC (H), FEV50 (I), MMEF (J), FEF50 (K), and FEF75 (L), were performed and compared between groups treated with and without IL-17A block. Data are representative of three independent experiments, and are presented as mean ± SD (n = 3 per group). *P < 0.05, **P < 0.01. COPD, chronic obstructive pulmonary disease; Cip, ciprofloxacin; CFU, colony-forming units; MPO, myeloperoxidase; FVC, forced vital capacity; FEV50, volume expired in the first 50 ms of fast expiration; MMEF, maximal mid-expiratory flow; FEF50, forced expiratory flow at 50% FVC; FEF75, forced expiratory flow at 75% FVC. [file Image_6.jpeg]
