# Supplementary material for: IL-17 Aggravates Pseudomonas aeruginosa Airway Infection in Acute Exacerbations of Chronic Obstructive Pulmonary Disease
Source: Front Immunol. 2022 Jan 13;12:811803. doi: 10.3389/fimmu.2021.811803 (PMC8792752; doi:10.3389/fimmu.2021.811803)
Supplement: Supplementary file 7 [file Table_1.docx]

**Appendices:**

Table S1. Primer sequences used for PCR

| Primers | Forward (5’→3’) | Reverse (5’→3’) |
| --- | --- | --- |
| mβ-Actin | 5' CCT CTA TGC CAA CAC AGT 3' | 5' AGC CAC CAA TCC ACA CAG 3' |
| mIL-23 | 5' CAA CTC TGA CTG AGC CCT TAG TG 3' | 5' ATA ATG GTG TCC TTG CCC TTC 3' |
| mIL-23R | 5' ATT CTT GAT GAA TTG TGC CTC 3' | 5' GTT TCA CTG GGT GAG TCA CTT 3' |
| mIL-17A | 5' GTT CGT GCT ATT GAT TTT CAG C 3' | 5' GGA CCC CTT TAC ACC TTC TTT 3' |
| mIL-17RA | 5' ACT TGG GAA CTG AGA CTT GAT 3' | 5' AAG GGG TGT GTT TAG GGA T 3' |
| IL-1β | 5' TAC ATC AGC ACC TCA CAA GC 3' | 5' AGA AAC AGT CCA GCC CAT ACT 3' |
| mCXCL1 | 5' CCA AGT AAC GGA GAA AGA AGA 3' | 5' TAG GAC CCT CAA AAG AAA TTG 3' |
| mCXCL15 | 5' CAA AGT CTT ATC CCA CTC CAC 3' | 5' CAA AGA AGT ATT TCA CCC TGG 3' |
| mIL-18 | 5' AAT GGA GAC CTG GAA TCA GAC 3' | 5' TTG TCA ACG AAG AGA ACT TGG 3' |
| mTNF-α | 5' TAG CCA GGA GGG AGA ACA GA 3' | 5' CCA GTG AGT GAA AGG GAC AGA 3' |
| mMMP9 | 5' CTG GAA CTC ACA CGA CAT CTT 3' | 5' CAC CTT GTT CAC CTC ATT TTG 3' |
| mIL-10 | 5' AGT GTG TAT TGA GTC TGC TGG 3' | 5' GAG AGA GGT ACA AAC GAG GTT 3' |
| mIL-1Ra | 5' TGA ATC CTG TGA CCC TGT G 3' | 5' AAA CTG AAC CCC TGA GAA GAG 3' |
| mTGF-β | 5' TGG TGG TGA AGT CGT GTA AGT G 3' | 5' TGA GGT CTG TCG CTT TGG TT 3' |
| mRBP4 | 5' TGG TGG GCA CTT TCA CAG ACA 3' | 5' TCA TCG TTT CCT CGC TGG AGA 3' |

m, mouse
